# A Global High-Resolution Precipitation Climate Record: PERSIANN-CCS-CDR Version 2.0

**DOI:** 10.1038/s41597-026-06625-5

**Published:** 2026-01-29

**Authors:** Mohammad Bolboli Zadeh, Phu Nguyen, Kuo-Lin Hsu, Amir AghaKouchak, Tu Thanh Ung, Soroosh Sorooshian

**Affiliations:** Center for Hydrometeorology and Remote Sensing, University of California, Irvine, USA

**Keywords:** Hydrology, Hydrology

## Abstract

PERSIANN-CCS-CDR is a global precipitation dataset with 0.04^°^ spatial and 3 hourly temporal resolution starting from 1983. However, producing reliable information proved to be a challenging task, primarily due to inconsistencies in input data (e.g., inconsistencies between GridSat-B1 and CPC-4km). Recognizing the existence of these inconsistencies and to address the issues, we introduce the new version of PERSIANN-CCS-CDR, called PERSIANN-CCS-CDR V2.0, the new version consists of two sub-products with the same spatial-temporal properties but different input data and record period: 1) PERSIANN-CCS-CDR-B1, which uses the GridSat-B1 as input and is available since 1983, and 2) PERSIANN-CCS-CDR-CPC, which uses the CPC-4KM as input and is available since March 2000. This paper highlights the limitations of the previous version of PERSIANN-CCS-CDR, introduces PERSIANN-CCS-CDR V2.0, evaluates its performance, and provides guidance for future users on how to effectively utilize the new product.

## Background & Summary

With the increasing frequency and intensity of extreme storms, the need for high-resolution global precipitation climate data records has become increasingly apparent. These trends, driven in part by climate change, highlight the importance of accurate and detailed precipitation monitoring. However, producing such a climate data record is a challenging task. Firstly, such a dataset requires a data source with a high spatial resolution, which makes gauge data, as the only source, an inadequate candidate. Furthermore, since constructing and maintaining weather radar networks comes with heavy financial burdens^1^, constructing a global network of weather radars is impractical, specifically when we take into account the massive economic, scientific, and bureaucratic gap between the Global South and North. As a result, satellite-based remote sensing remains the only viable option for consistent global precipitation monitoring. Although there are plenty of options to choose from when it comes to satellite data, unfortunately, each comes with its shortcomings. Satellites can be divided into two main categories: Geostationary Earth Orbiting (GEO) and Low Earth Orbiting (LEO). LEOs have the advantage of having passive microwave (PMW) sensors on board, which provide more physics-based information that can be directly translated to precipitation; however, LEO’s data is infrequent, specifically in mid-latitudes^2–6^. On the other hand, GEOs provide frequent infrared (IR) samples with almost global coverage; however, they can only provide information on cloud-top characteristics^2,7^, which is not as accurate an indicator of precipitation as PMW data. While LEO satellites are valuable for global precipitation monitoring, their infrequent sampling poses challenges for capturing short-duration, high-intensity events. As such, their current spatial and temporal resolution may limit their ability to fully resolve extreme storms. Hence, GEO’s IR data offer a unique opportunity to produce global precipitation datasets with the spatial-temporal resolution needed to better capture extreme events, a great example of such effort is the Climate Hazards group Infrared Precipitation with Stations (CHIRPS) dataset^8^. Currently, there are two main global GEO’s IR datasets available. The first one is the National Oceanic and Atmospheric Administration’s (NOAA) Climate Prediction Center (CPC) globally merged IR product at 4 km and 30-minute temporal resolution (CPC-4km)^9,10^. The second one is the NOAA’s Gridded Satellite B1 data (GridSat-B1) globally merged dataset with 3-hour temporal resolution and 0.07^°^ spatial resolution^10,11^. The former is the best candidate for input into a global dataset with high temporal and spatial resolution. However, since it is only available starting from March 2000, the record is not sufficiently long to be suitable for a climate data record. The latter, despite having a long record period, lacks the spatial-temporal resolution required for producing a high-resolution product. Therefore, given the current state of available inputs, the only feasible approach to creating a global, high-resolution precipitation climate data record is to somehow leverage the strengths of both datasets. Overcoming this hurdle was Precipitation Estimation from Remotely Sensed Information using Artificial Neural Networks-Cloud Classification System-Climate Data Record’s (PERSIANN-CCS-CDR) biggest goal.

The Center for Hydrometeorology and Remote Sensing (CHRS) at the University of California, Irvine (UCI) has a rich history of producing precipitation estimation products using satellite imagery^2,12–15^. Although satellite data have their limitations, CHRS has continuously produced high-skill products that are well-trusted among the scientific community, most notably Precipitation Estimation from Remotely Sensed Information using Artificial Neural Networks - Climate Data Record (PERSIANN-CDR)^15^ and Precipitation Estimation from Remotely Sensed Information using Artificial Neural Networks - Dynamic Infrared Rain Rate near real-time (PDIR-Now)^2^. On top of that, Precipitation Estimation from Remotely Sensed Information using Artificial Neural Networks - Cloud Classification System (PERSIANN-CCS)^14^ algorithm is one of the core components that contribute to NASA’s Integrated Multi-satellitE Retrievals for GPM (IMERG)^16^. In 2021, CHRS published a new product, PERSIANN-CCS-CDR^17^: a “near-global 37+ year high-resolution precipitation dataset with both high spatial and temporal resolutions’^18^ intending to solve a major challenge: to produce a high spatial and temporal resolution climate data record that uses two different inputs: the GridSat-B1 from 1983 to 2000 and CPC-4km from March 2000 onward^17^. PERSIANN-CCS-CDR received (and continues to receive) a lot of enthusiasm from the community, which further emphasizes the need for such a dataset.

**Fig. 1:**
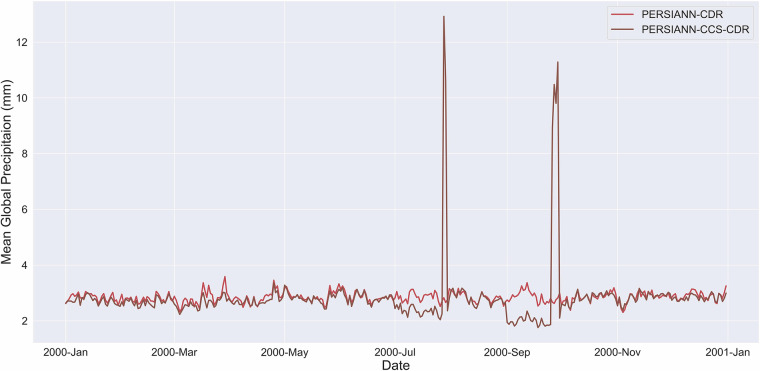
The daily mean global precipitation in 2000 for PERSIANN-CDR and PERSIANN-CCS-CDR. The figure shows how only a few corrupt files can result in significant errors for the entire month, as indicated by the noticeable dip preceding the surges.

Unfortunately, PERSIANN-CCS-CDR faced a series of challenges shortly after its release. Firstly, PERSIANN-CCS-CDR was mistakenly selected by some users for the wrong applications, which led to the poor performance of PERSIANN-CCS-CDR in their analysis. PERSIANN-CCS-CDR was a tool specifically developed for tasks that required high spatial-temporal resolutions. It was never supposed to provide the same level of performance at lower resolutions or tasks that didn’t require a high spatial-temporal resolution. PERSIANN-CCS-CDR was not an upgrade to the already existing catalog of CHRS products (i.e., PERSIANN-CDR), but instead, it was an addition to it. Furthermore, PERSIANN-CCS-CDR suffered from multiple issues. One issue was that the product contained numerous corrupt files, which were only identified after a thorough investigation. Due to the way that PERSIANN-CCS-CDR is bias corrected according to the Global Precipitation Climatology Project (GPCP) Monthly Analysis Product^17^, the impact of these corrupt files reached far beyond the files themselves. For example, Fig. 1 shows the daily mean global precipitation of PERSIANN-CDR and PERSIANN-CCS-CDR for the year 2000. From the figure, it is evident how only a few corrupt files can result in significant errors in the entire month, as indicated by the noticeable dip preceding the surges. Unfortunately, the replacement of these corrupt files did not solve PERSIANN-CCS-CDR’s main issue.

**Fig. 2:**
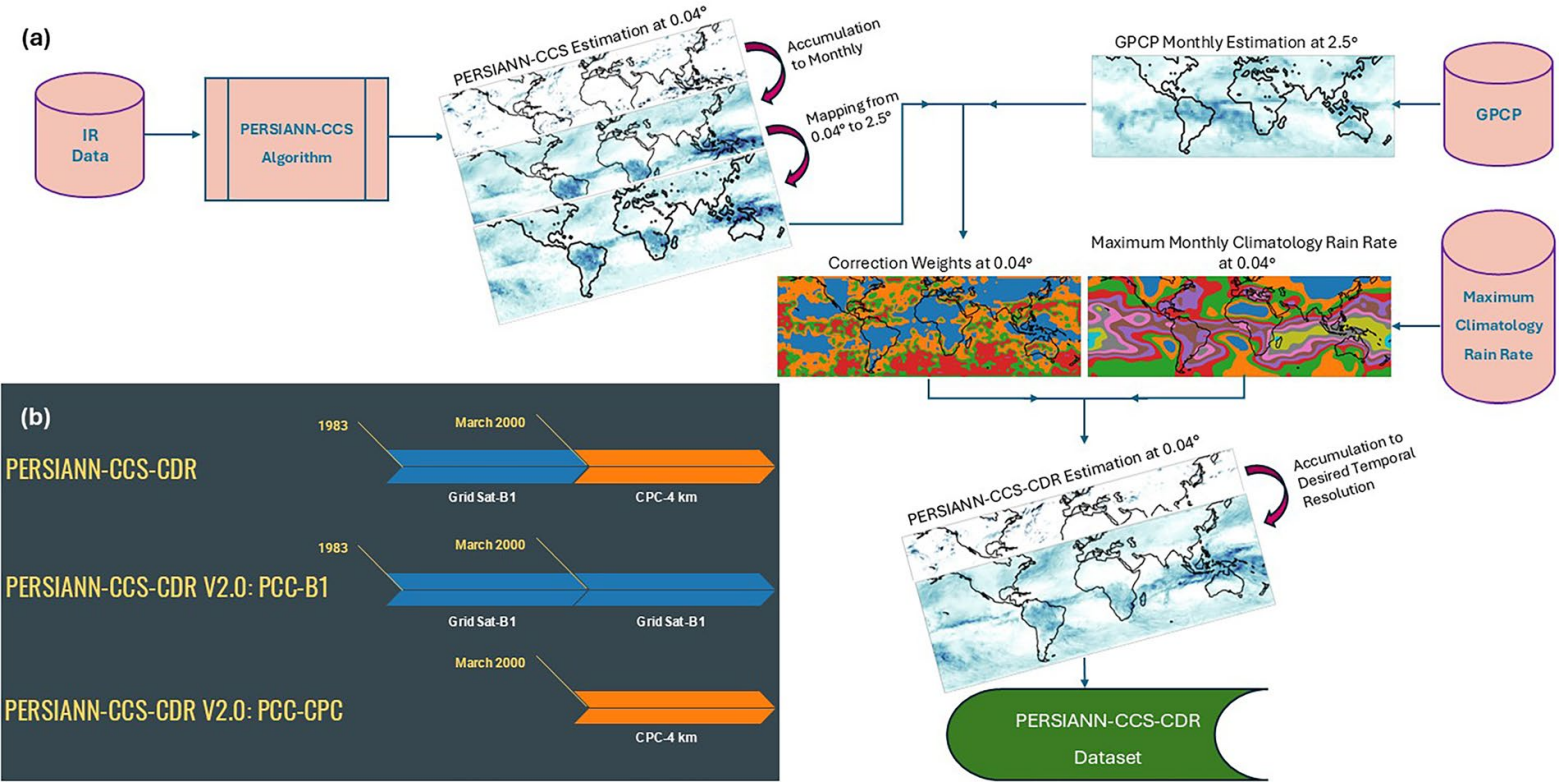
Flow chart of PERSIANN-CCS-CDR process (**a**) and overview of different versions of PERSIANN-CCS-CDR and their input time line (**b**).

An in-depth analysis of the dataset revealed significant inconsistencies in PERSIANN-CCS-CDR starting in the year 2000, a year in which the PERSIANN-CCS-CDR input changes from GridSat-B1 to CPC-4km. These inconsistencies were the reason for the withholding of PERSIANN-CCS-CDR from the CHRS data portal. Despite efforts to correct these inconsistencies, no breakthroughs could be reached, suggesting that the initial assumption of successfully merging the GridSat-B1 and CPC-4km datasets may have been overly optimistic. To address these inconsistencies, CHRS chose to release two consistent sub-products under a single parent product. The new revision of PERSIANN-CCS-CDR, named PERSIANN-CCS-CDR Version 2.0^19^, consists of two sub-products: 1) PERSIANN-CCS-CDR Version 2.0 B1 (PERSIANN-CCS-CDR-B1), which is available since 1983 and uses GridSat-B1 as input, and PERSIANN-CCS-CDR Version 2.0 CPC (PERSIANN-CCS-CDR-CPC), which is available since March 2000 and uses the CPC-4km as input. Figure 2 shows a flow chart of the PERSIANN-CCS-CDR process and an overview of different versions of PERSIANN-CCS-CDR and their input time line.

The following sections focus on evaluating the strengths, weaknesses, and applications of these two datasets. This evaluation involves comparing the performance of these two products against the NCEP/EMC 4KM Gridded Data (GRIB) STAGE IV Data (STAGE IV)^20^ and PERSIANN-CDR. The PERSIANN-CCS-CDR-B1 and PERSIANN-CCS-CDR-CPC datasets are evaluated from two perspectives: first, as a precipitation climate data record, by examining their performance on precipitation extreme indices; and second, as a high-resolution precipitation record, by assessing their performance during extreme events.

## Methods

### Region of Study

**Fig. 3:**
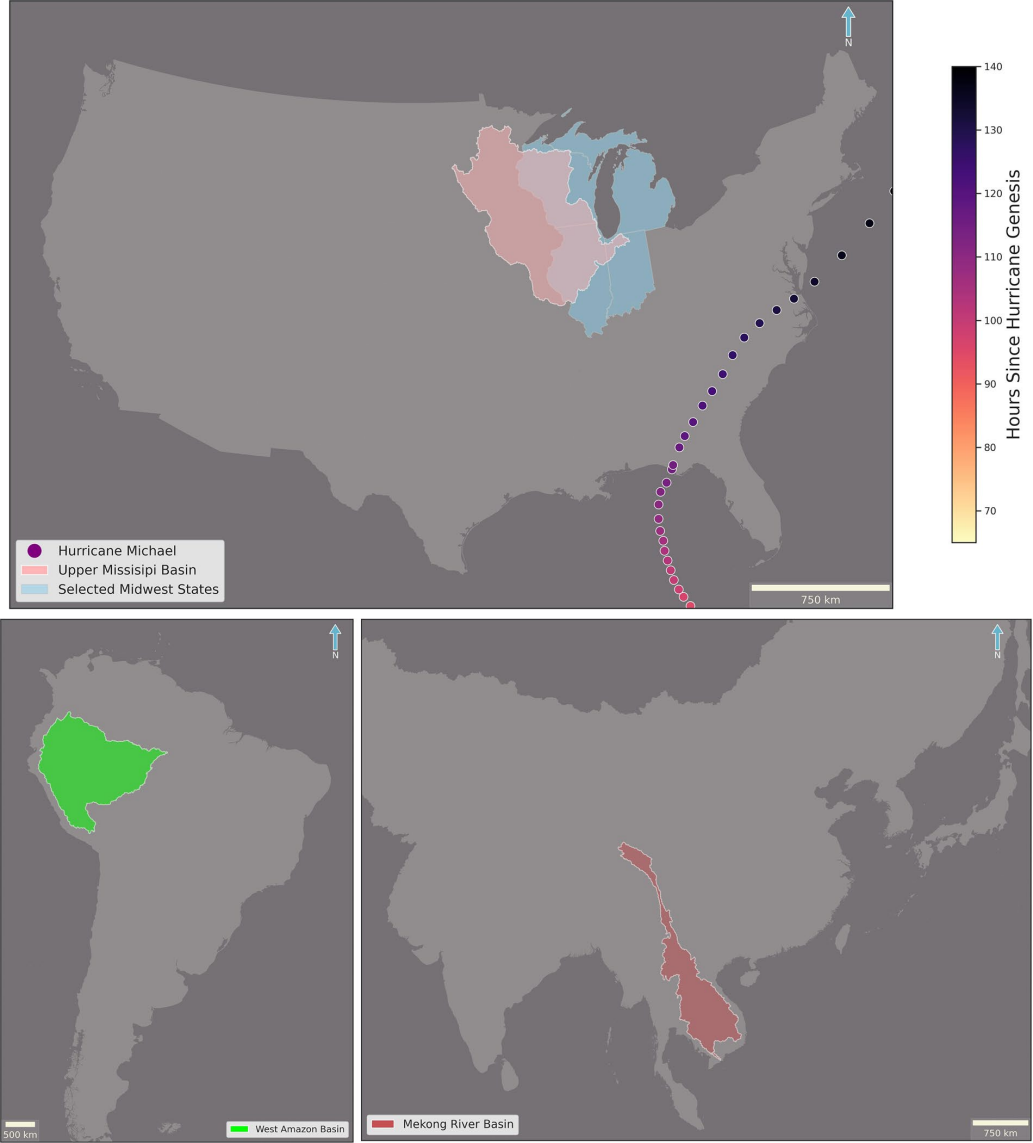
Region of Study:The top panel shows the track of Hurricane Michael in circles, colored by hours since its genesis. The Upper Mississippi Basin is highlighted in pink, and selected Midwest states are highlighted in blue. The bottom-left panel shows the West Amazon basin in green, while the bottom-right panel shows the Mekong River basin in red.

Although both the PERSIANN-CCS-CDR-CPC and PERSIANN-CCS-CDR-B1 products have a near-global coverage^2,12,14,15,17^ because one of the objectives of this study was to compare them with the STAGE IV product, the region of the study for intercomparison was limited to CONtiguous United States (CONUS) where STAGE IV data is publicly available. Furthermore, for the first part of the study concerning extreme precipitation climate indices, the upper Mississippi basin (Fig. 3) was chosen because it satisfied two main criteria: firstly, it was in Eastern CONUS, as STAGE IV has poor coverage and performance in Western CONUS compared to Eastern CONUS^21–24^. And secondly, the selected basin’s area is large enough to ensure meaningful results, yet not so large that averaging would suppress the signal. To evaluate the performance of the datasets during extreme events, we first looked at Hurricane Michael, which was a Category 5 hurricane that made landfall in October 2018. On top of that, the datasets were evaluated during the July 2024 Upper Midwest severe weather outbreak. This event produced hurricane-force winds, multiple tornadoes, and fast-moving showers, and it caused widespread power outages and was classified by NOAA as a billion-dollar weather and climate disaster^25,26^. The path for Hurricane Michael, along with the location of the upper Mississippi basin, and selected Midwest states for July 2024 event can be seen in Fig. 3. Additionally, to further demonstrate the consistency of the new dataset across different regions and climates, a similar analysis of extreme precipitation climate indices was conducted over the West Amazon Basin and the Mekong River Basin. The locations of these selected basins are shown in Fig. 3.

### Data Sources

#### PERSIANN-CDR

PERSIANN-CDR is a near-global dataset with coverage over 60^°^ South to 60^°^ North and 180^°^ West and 180^°^ East from January 1983 until the present^15^. And it has stood the test of time regarding its performance and consistency. It takes the GridSat-B1 data as input and uses the Precipitation Estimation from Remotely Sensed Information using Artificial Neural Networks (PERSIANN) algorithm to estimate rainfall at 0.25^°^ by 0.25^°^ spatial resolution and is available in daily, monthly, and yearly time resolutions^12,15^. In this study, the daily product is used.

#### PERSIANN-CCS-CDR-B1

The PERSIANN-CCS-CDR-B1 is a near-global dataset with coverage over 60^°^ South to 60^°^ North and 180^°^ West and 180^°^ East from January 1983 until the present. It takes the GridSat-B1 data as input, which is available every three hours, to estimate precipitation with 0.04^°^ by 0.04^°^ spatial resolution and is available in 3-hourly, 6-hourly, daily, monthly, and yearly time resolutions. In this study, the 3-hourly and daily products are used.

#### PERSIANN-CCS-CDR-CPC

The PERSIANN-CCS-CDR-CPC dataset has identical properties concerning spatial coverage, spatial resolution, and temporal resolution as the PERSIANN-CCS-CDR-B1 dataset, and it uses the same algorithm. What sets it apart from the PERSIANN-CCS-CDR-B1 dataset is that, firstly, it uses the CPC-4km product as input, which is available every 30 minutes. And secondly, the shared algorithm between the PERSIANN-CCS-CDR-B1 and PERSIANN-CCS-CDR-CPC datasets is tailored to fit the CPC-4KM data. Also, since the PERSIANN-CCS-CDR-CPC uses the CPC-4km data as input, it’s only available starting in March 2000.

#### STAGE IV

The STAGE IV dataset incorporates both the radar and gauge data to estimate hourly, 6-hourly, and 24-hour precipitation at roughly 4 km resolution over the CONUS^20^. In this study, the hourly product was used, which was then accumulated to 3-hourly and daily.

#### International Best Track Archive for Climate Stewardship (IBTrACS)

The IBTrACS is the gold standard when it comes to historical and recent tropical cyclonic best-track data^27,28^. It combines the data from various agencies to produce a single best-track database for tropical cyclones^27,28^.

### Data Preparation

Shape files provided by^29,30^ were utilized to subset the datasets to CONUS, Upper Mississippi basin, West Amazon basin, and Mekong River Basin. Since STAGE IV, PERSIANN-CCS-CDR-CPC, and PERSIANN-CCS-CDR-B1 datasets have a 0.04^°^ resolution, and PERSIANN-CDR has a 0.25^°^ resolution, in instances where PERSIANN-CDR was included in comparisons, other datasets were mapped to 0.25^°^ resolution using a bilinear interpolation.

## Data Records

PERSIANN-CCS-CDR V2.0^19^ is a high spatial-temporal resolution precipitation estimation dataset developed at CHRS at UCI that consists of two sub-products: PERSIANN-CCS-CDR-CPC, available from March 2000, and PERSIANN-CCS-CDR-B1, available from 1983. Both datasets have a 0.04^°^ spatial and 3-hourly temporal resolution over the global domain of 60^°^S to 60^°^N and are available publicly through the CHRS Data Portal^12^ (https://chrsdata.eng.uci.edu/) at 3-hourly, 6-hourly, daily, monthly, and yearly time steps in ArcGrid, Tif, and NetCDF format. The data is also available through the CHRS FTP website (https://persiann.eng.uci.edu/CHRSdata/PCCSCDR_B1/) and https://persiann.eng.uci.edu/CHRSdata/PCCSCDR_CPC/) in binary format. The final PERSIANN-CCS-CDR V2.0 rainfall data are provided as accumulated precipitation (in mm) at 3-hourly, 6-hourly, daily, monthly, and yearly intervals, each stored in separate directories. File names indicate both the accumulation period and the start time. For instance, in the file name PCCSCDRXXYYMMDDhh.bin.gz,

- XX represents the accumulation period (e.g., 3 h, 6 h, 1 d, 1 m, 1 y).
- YYMMDD denotes the year, month, and day.
- hh indicates the starting hour of the accumulation period.

For example, PCCSCDR3h17010103.bin.gz represents rainfall accumulated over a 3-hour period on January 1, 2017, starting at 03:00Z and ending at 05:59Z. All data are in flat binary files 4-byte float (float32) values in little-endian byte-order. The area covered by dataset coresponds to 3000 rows  × 9000 columns. Data are stored in the file in row-major orientation with the first 9000 values the N-most row centered at 59.98N with longitude centers from .02 to 359.98 and the last row (S-most row) centered at 59.98S. For user convenience, the directory structure remains consistent with the previous version of PERSIANN-CCS-CDR (introduced in^17^), but with updated data files. Detailed documentation on data formats is available through the CHRS Data Portal and FTP site.

## Technical Validation

### Extreme Precipitation Climate Indices Methodology

To evaluate the performance of each dataset as a climate data record, eight extreme precipitation climate indices according to^31,32^ seen in Table 1 were calculated at the pixel level for the upper Mississippi basin: annual wet-day precipitation (PRCPTOT), very wet days (R95PTOT), extremely wet days (R99PTOT), simple daily precipitation index (SDII), and maximum 10-day precipitation (R10TOT) to assess intensity; heavy precipitation days (R10) to assess frequency; and consecutive dry days (CDD), and consecutive wet days (CWD) to assess duration. Firstly, since the previous version of PERSIANN-CCS-CDR suffered from inconsistencies in 2000, this year was chosen as a point for consistency assessment of datasets. Secondly, the mentioned precipitation extreme indices for PERSIANN-CCS-CDR-CPC and PERSIANN-CCS-CDR-B1 at both 0.25^°^ and 0.04^°^ resolutions and for PERSIANN-CDR at 0.25^°^ were compared to STAGE IV at 0.04^°^ and 0.25^°^ to assess the performance of each dataset.

**Table 1:** Extreme precipitation climate indices used in this study.

Index	Index Name	Index Definitions	Units
PRCPTOT	Annual wet-day precipitation	Annual total precipitation in wet days	mm
R95PTOT	Very wet days	Annual total precipitation from days greater than the 95^th^ percentile	mm
R99PTOT	Extremely wet days	Annual total precipitation from days greater than the 99^th^ percentile	mm
R10TOT	Max 10-day precipitation	Annual maximum precipitation over a 10-day period	mm
R10	Heavy precipitation days	Annual count of days when precipitation exceeds 10 mm	days
SDII	Simple daily precipitation index	The ratio of annual total precipitation to the number of wet days	mm/day
CDD	Consecutive dry days	Maximum number of consecutive dry days	days
CWD	Consecutive wet days	Maximum number of consecutive wet days	days

### Extreme Events Methodology

To evaluate each dataset as a high-resolution precipitation record, their performance during extreme events (Hurricanes) was assessed. To address this, it was essential to adopt a methodology capable of thoroughly capturing the temporal and spatial aspects of hurricanes. To do so, at each time step, a box around the hurricane center was selected. The center of the box was extracted from the latitude and longitude variables of IBTrACS, and the radius of the storm at each time step was calculated as the average of the USA_R34_NW, USA_R34_NE, USA_R34_SE, and USA_R34_SW variables in IBTrACS. For daily analysis, the maximum radius during the event was selected as the storm radius. However, it was observed that selecting a box around the hurricane only as big as the storm radius failed to fully capture the extent of the hurricane’s impact. Hence, the dimensions of the box were quadrupled in each direction. In the case of daily analysis, at each time interval, multiple storm centers existed; therefore, the box was selected in a way that the above criteria would be satisfied for all the points. Furthermore, due to the complex nature of the problem, no single statistical test could tell the whole story. Therefore, the datasets were assessed using visual assessment, scatter plots, Root Mean Squared Error (RMSE), Correlation Coefficient (C.C.), percentile values, pixel count, Probability of Detection (POD), False Alarm Ratio (FAR), and Critical Success Index (CSI).

### Consistency Assessment of Extreme Precipitation Climate Indices

**Fig. 4:**
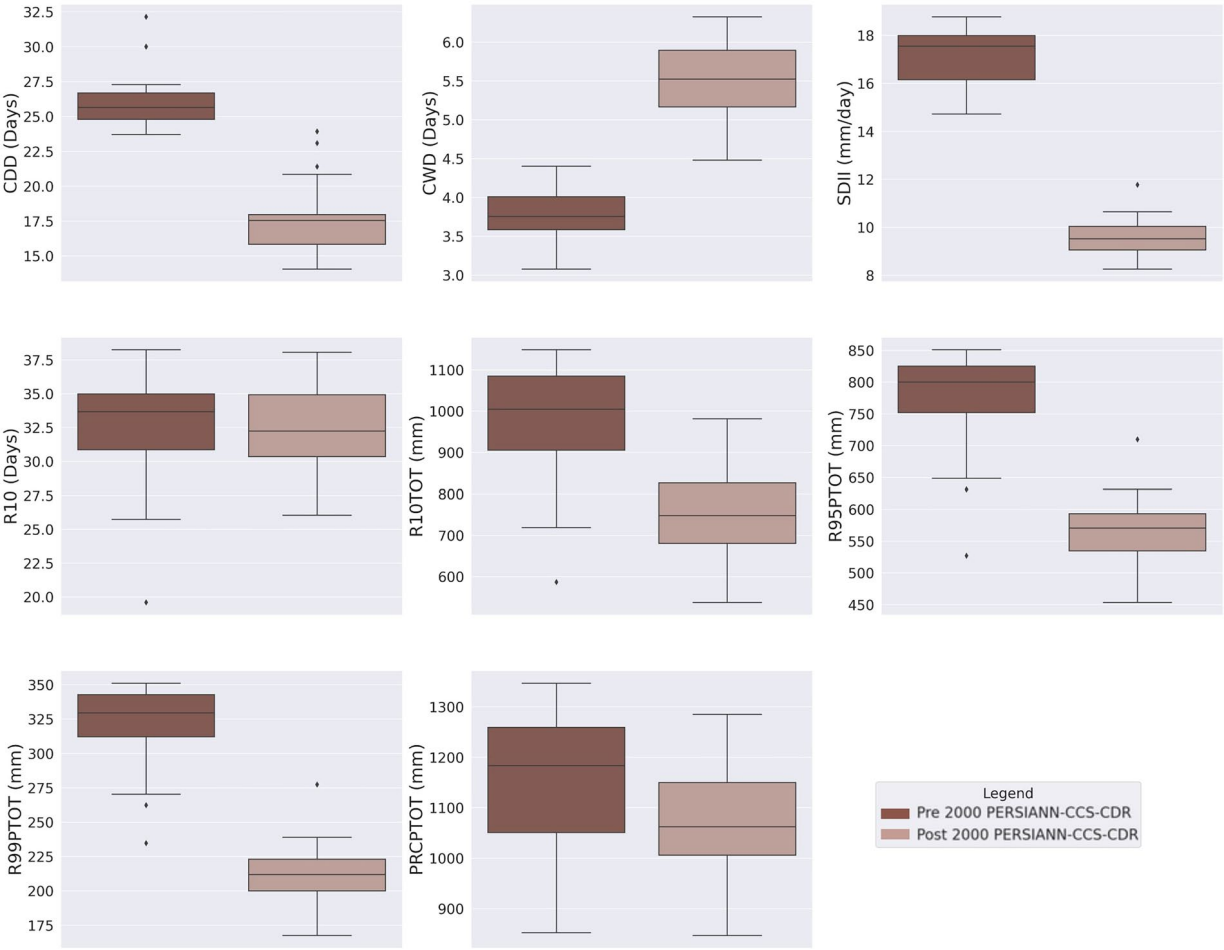
Box plot of mean yearly extreme precipitation climate indices for pre-2000 (1983–2000) in dark shade and post-2000 (2000–2019) in light shade based on the previous version of PERSIANN-CCS-CDR, at 0.04^°^ resolution for the upper Mississippi basin.

**Fig. 5:**
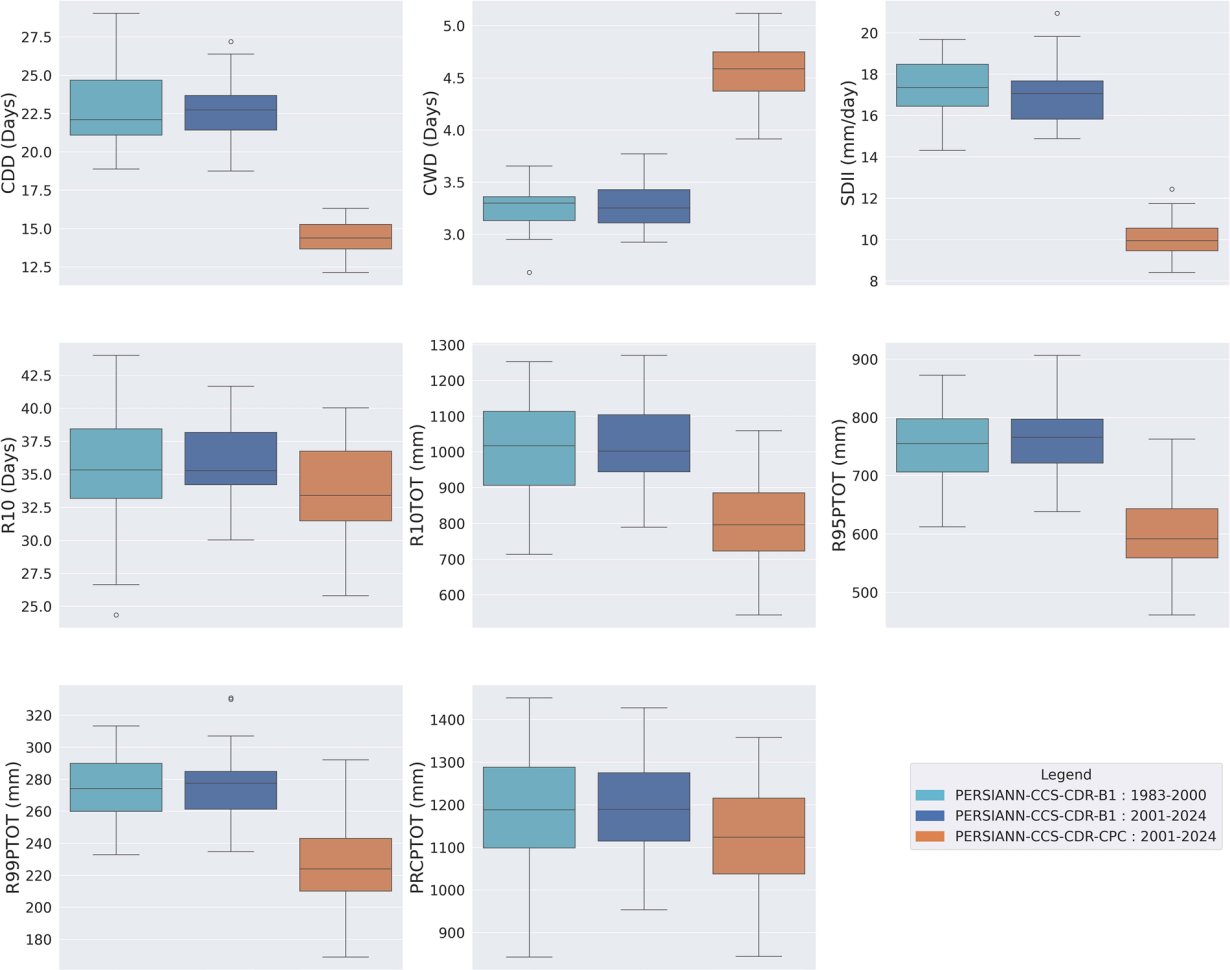
Box plots of mean yearly extreme precipitation climate indices for 1983–2000 PERSIANN-CCS-CDR-B1 in light blue, 2001–2024 PERSIANN-CCS-CDR-B1 in dark blue, and 2001–2024 PERSIANN-CCS-CDR-CPC in orange, at 0.04^°^ resolution for the upper Mississippi basin.

**Fig. 6:**
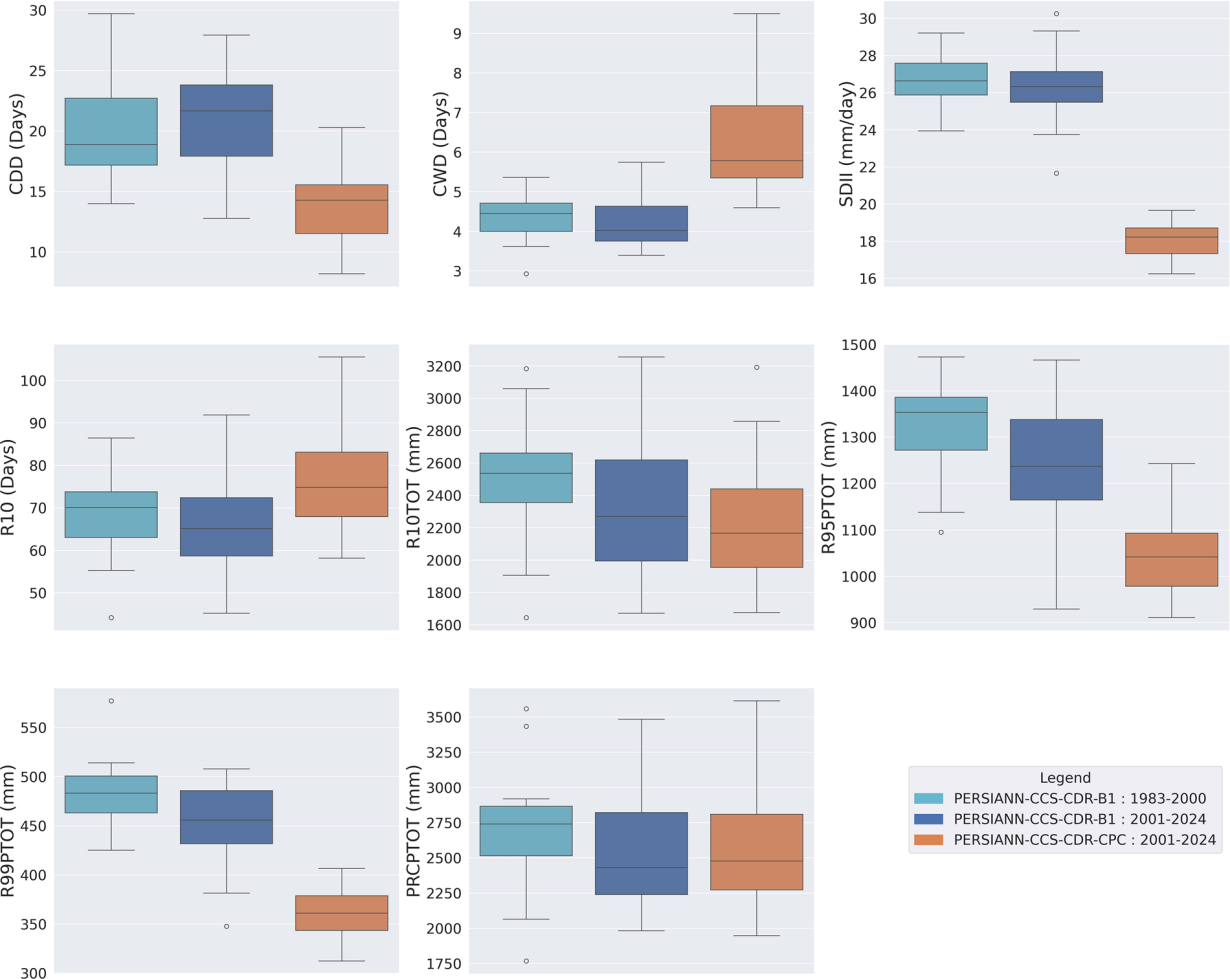
Box plots of mean yearly extreme precipitation climate indices for 1983–2000 PERSIANN-CCS-CDR-B1 in light blue, 2001–2024 PERSIANN-CCS-CDR-B1 in dark blue, and 2001–2024 PERSIANN-CCS-CDR-CPC in orange, at 0.04^°^ resolution for the west Amazon basin.

**Fig. 7:**
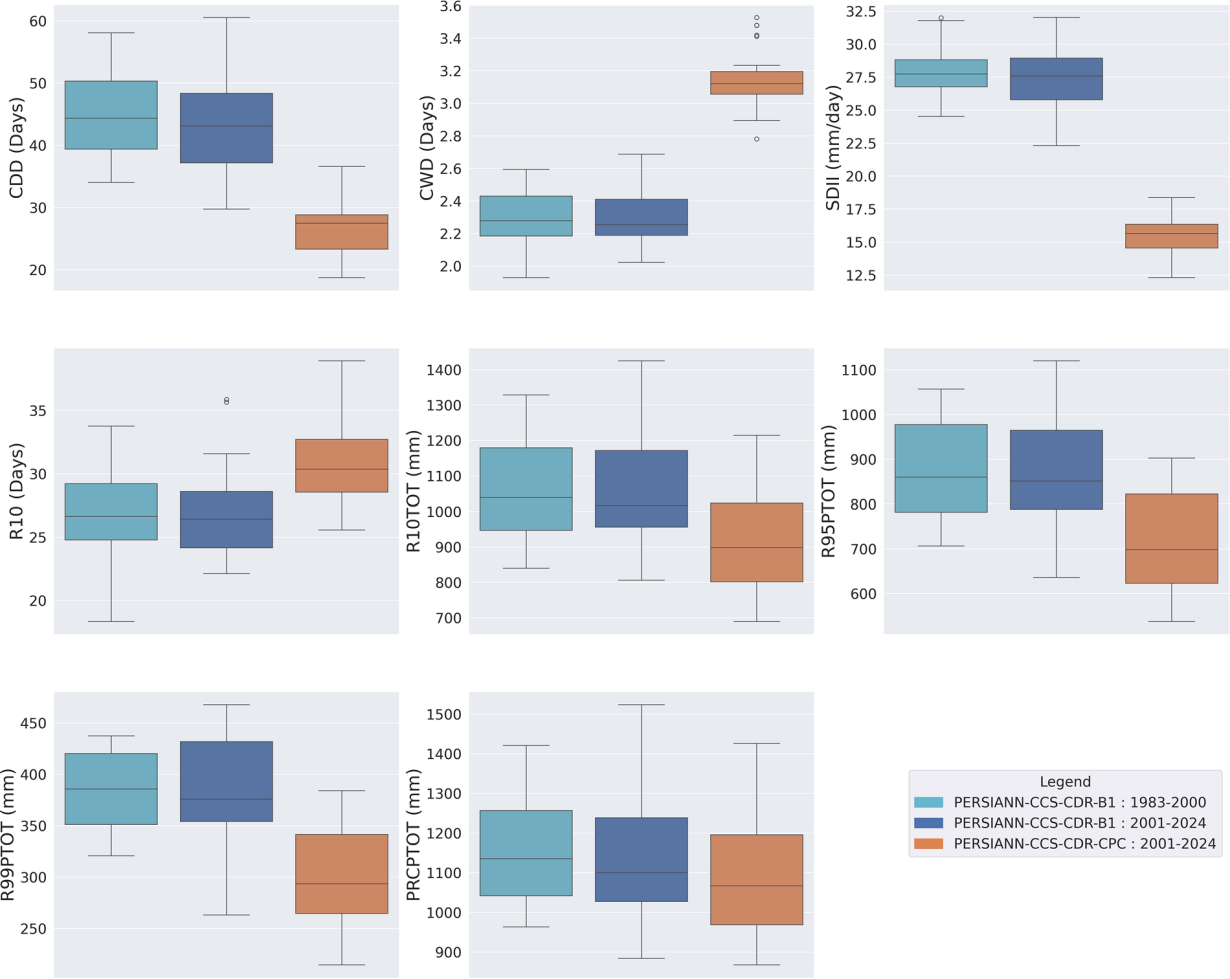
Box plots of mean yearly extreme precipitation climate indices for 1983–2000 PERSIANN-CCS-CDR-B1 in light blue, 2001–2024 PERSIANN-CCS-CDR-B1 in dark blue, and 2001–2024 PERSIANN-CCS-CDR-CPC in orange, at 0.04^°^ resolution for the The Mekong River basin.

As mentioned earlier, the previous version of PERSIANN-CCS-CDR suffered from major inconsistencies, which are easily visible through extreme precipitation climate indices. Figure 4 shows the box plot of mean yearly extreme precipitation climate indices for pre-2000 (1983–2000) and post-2000 (2000–2019) periods based on the previous version of PERSIANN-CCS-CDR at 0.04^°^ resolution for the upper Mississippi basin. From the figure, it is clear that, except for R10 and PRCPTOT, all other indices suffer from major inconsistencies at the break line of the year 2000, which is the year that the model input changes from GridSat-B1 to CPC-4km. To address this issue, CHRS decided to publish a new version of PERSIANN-CCS-CDR, consisting of two separate datasets. Figure 5 Shows the box plot of mean yearly extreme precipitation climate indices for 1983–2000 and 2001–2024 PERSIANN-CCS-CDR-B1 and 2001–2024 PERSIANN-CCS-CDR-CPC, at 0.04^°^ resolution for the upper Mississippi basin. From the figure, it’s clear that in PERSIANN-CCS-CDR V2.0, the inconsistencies have been resolved. However, it’s also evident that for the 2001–2024 period, the performance of PERSIANN-CCS-CDR-B1 and PERSIANN-CCS-CDR-CPC is very different, raising the need to investigate which of these two datasets has a better performance. As mentioned earlier, to further demonstrate the consistency of the new dataset across different regions and climates, a similar analysis of extreme precipitation climate indices was conducted over the West Amazon Basin and the Mekong River Basin. Figure 6 Shows the box plot of mean yearly extreme precipitation climate indices for 1983–2000 and 2001–2024 PERSIANN-CCS-CDR-B1 and 2001–2024 PERSIANN-CCS-CDR-CPC, at 0.04^°^ resolution for the west Amazon basin, and Fig. 7 shows the same plot for the Mekong River basin. Both of these plots demonstrate the same point as Fig. 5, that PERSIANN-CCS-CDR V2.0 is a consistent dataset, however, its different sub products perform significantly different from each other.

### Performance Assessment of Extreme Precipitation Climate Indices

**Fig. 8:**
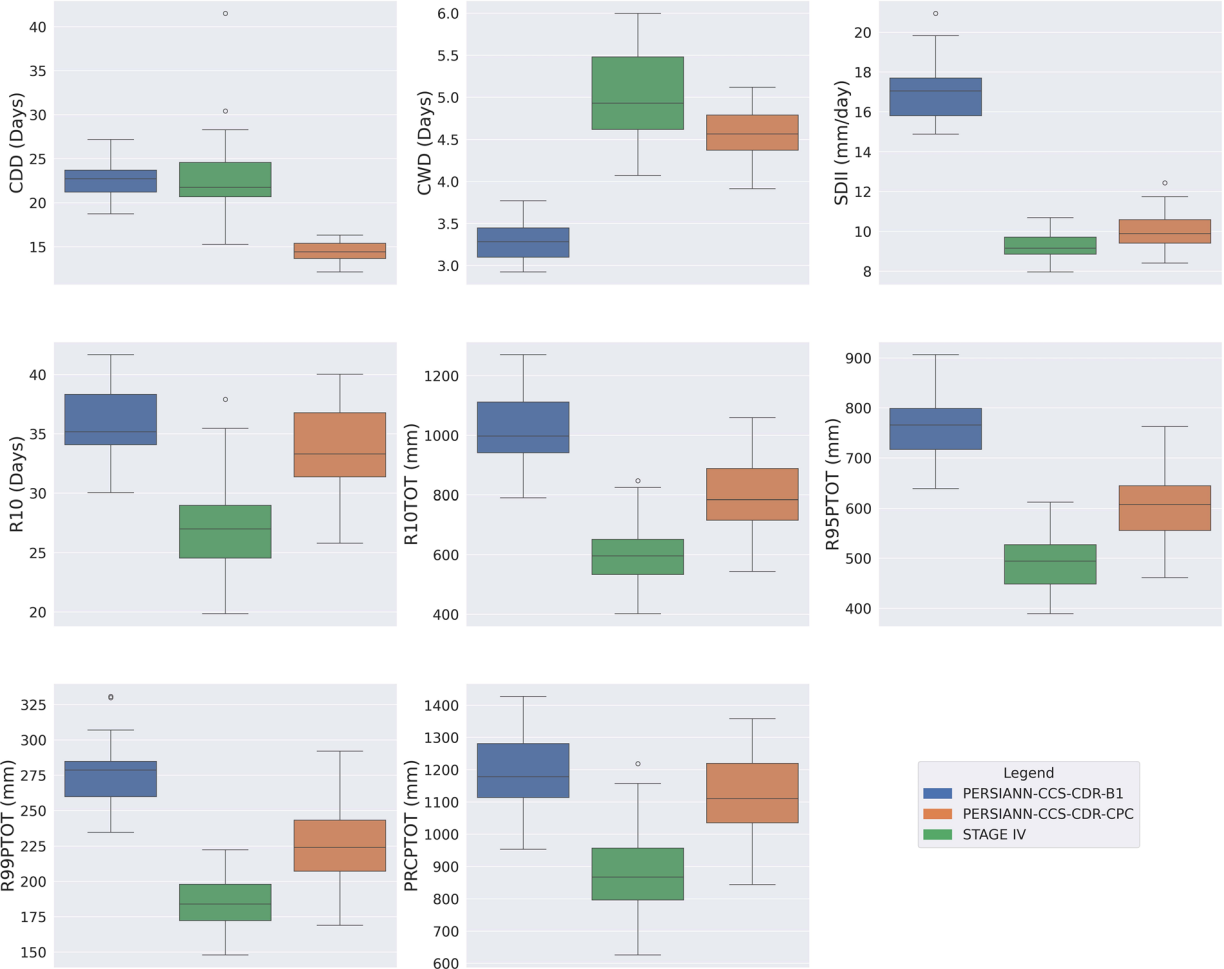
Box plots of mean yearly extreme precipitation climate indices for PERSIANN-CCS-CDR-B1 in blue, PERSIANN-CCS-CDR-CPC in orange, and STAGE IV in green for the upper Mississippi basin at 0.04^°^ resolution for the period of 2002–2023.

To assess the performance of PERSIANN-CCS-CDR-B1 and PERSIANN-CCS-CDR-CPC, their extreme precipitation climate indices (Table 1) were compared to that of STAGE IV at 0.04^°^ resolution for the upper Mississippi basin for the period of 2002–2023. Figure 8 shows the box plot of mean values of extreme precipitation climate indices for this period. Although both datasets have a close performance to STAGE IV, for almost all the indices, PERSIANN-CCS-CDR-CPC has a closer performance to STAGE IV, with the only exception being the CDD. The RMSE values for mean values of extreme precipitation climate indices for PERSIANN-CCS-CDR-CPC and PERSIANN-CCS-CDR-B1 can be seen in Table 2.

**Table 2:** RMSE values for PERSIANN-CCS-CDR-CPC and PERSIANN-CCS-CDR-B1 compared to STAGE IV for extreme precipitation climate indices at 0.04^°^ resolution for the upper Mississippi basin for the period of 2002–2023.

Index	RMSE for PERSIANN-CCS-CDR-CPC	RMSE for PERSIANN-CCS-CDR-B1
CDD (Days)	10.2	5.6
CWD (Days)	0.6	1.8
SDII (mm/day)	1.2	7.8
R10 (Days)	7.1	8.9
R10TOT (mm)	221.4	429.4
R95PTOT (mm)	122.4	275.6
R99PTOT (mm)	49.0	94.6
PRCPTOT (mm)	260.4	325.8

It is also worth mentioning that PERSIANN-CCS-CDR-CPC performs best regarding SDII, R99PTOT, and R95PTOT, which are all intensity-related indices and are aligned with the original goal of PERSIANN-CCC-CDR to capture high intensity events. On the other hand, PERSIANN-CCS-CDR-B1 outperforms PERSIANN-CCS-CDR-CPC in the CDD index. However, this advantage is not due to its superior skill. Instead, it is primarily an artifact of PERSIANN-CCS-CDR-B1’s lower temporal resolution, which naturally reduces the likelihood of capturing events, and secondarily a result of issues in PERSIANN-CCS-CDR-B1’s downsampling process, which also leads to PERSIANN-CCS-CDR-B1’s poor performance in the CWD and SDII indices. This problem causes PERSIANN-CCS-CDR-B1 to concentrate most of the rainfall in a few pixels while leaving most other pixels dry. This issue will be discussed in more detail in the following chapters. Concerning PRCPTOT, both datasets have similar performance, as both of them are bias-corrected based on GPCP.

**Fig. 9:**
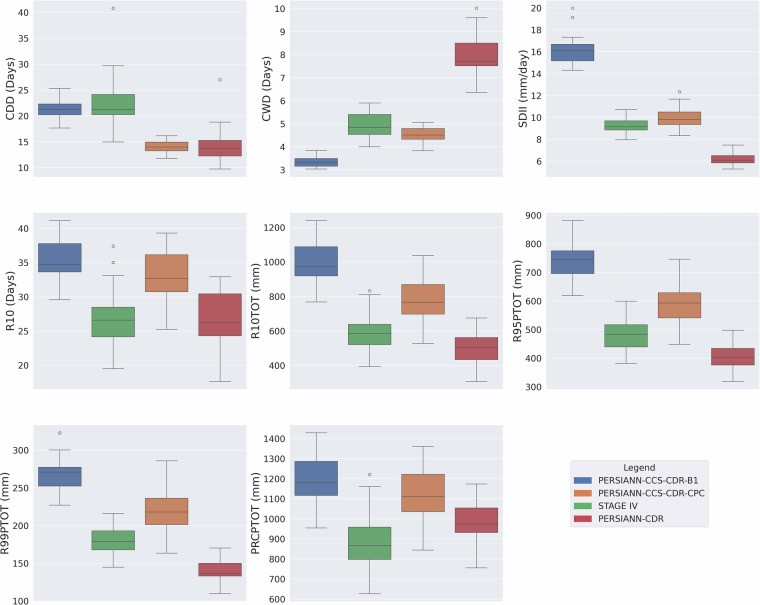
Box plots of mean yearly extreme precipitation climate indices for PERSIANN-CCS-CDR-B1 in blue, PERSIANN-CCS-CDR-CPC in orange, PERSIANN-CDR in red, and STAGE IV in green for upper Mississippi basin at 0.25^°^ resolution for the period of 2002–2023.

To further explore the performance of datasets, we extended our analysis to 0.25^°^ resolution with the inclusion of the PERSIANN-CDR dataset. Figure 9 presents these findings, highlighting similar patterns observed at 0.04^°^ resolution. PERSIANN-CCS-CDR-B1 has the best performance compared to STAGE IV in CDD due to previously explained reasons. PERSIANN-CCS-CDR-CPC has the best performance compared to STAGE IV in CWD, SDII, R10TOT, R95PTOT, R99PTOT, and PERSIANN-CDR has the best performance in R10 and PRCPTOT. From the figure we can conclude that when it comes to intensity indices, PERSIANN-CCS-CDR-CPC has the best performance in four (SDII, R10TOT, R95PTOT, and R99PTO) out of five of these indices. This further demonstrates how effective PERSIANN-CCS-CDR-CPC is in its intended purpose. Comparing PERSIANN-CDR with PERSIANN-CCS-CDR-B1 shows that PERSIANN-CDR performs comparably to PERSIANN-CCS-CDR-B1 across nearly all indices. However, PERSIANN-CDR (as we will see in the next sections) is unable to capture extreme precipitation values due to its low resolution. Another point worth mentioning is that PERSIANN-CDR, despite its old algorithm, generally outperforms PERSIANN-CCS-CDR-B1 and also has superior performance in PRCPTOT than both PERSIANN-CCS-CDR-B1 and PERSIANN-CCS-CDR-CPC, compared to STAGE IV, which shows how reliable PERSIANN-CDR is at lower resolutions.

### Performance Assessment During Extreme Events

#### Hurricane Michael Daily Analysis at 0.04^°^ Resolution

This section provides the results from the testing and evaluation of PERSIANN-CCS-CDR-B1 and PERSIANN-CCS-CDR-CPC during extreme events. To achieve this, PERSIANN-CCS-CDR-B1 and PERSIANN-CCS-CDR-CPC are first evaluated at a daily resolution, this approach allows us to concentrate on the datasets’ performance at a high spatial resolution by reducing the emphasis on temporal resolution. In the next step, the analysis is conducted at a 3-hour resolution to incorporate temporal resolution into the evaluation.

**Fig. 10:**
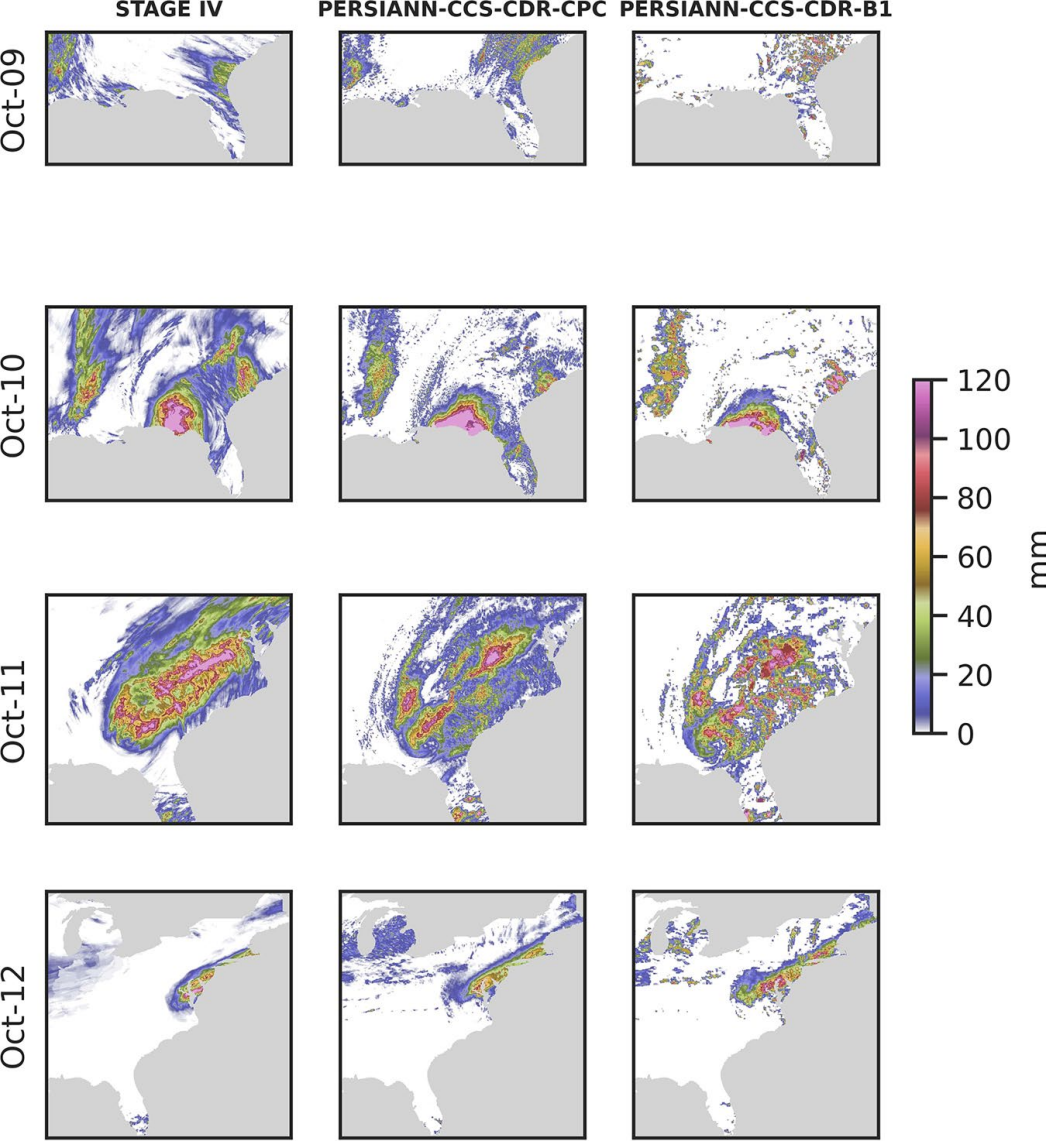
Snapshots of Hurricane Michael according to STAGE IV, PERSIANN-CCS-CDR-CPC, and PERSIANN-CCS-CDR-B1 dataset at 0.04^°^ and daily resolution.

Figure 10 shows the snapshots of Hurricane Michael captured by STAGE IV, PERSIANN-CCS-CDR-CPC, and PERSIANN-CCS-CDR-B1 datasets starting October 9^th^ until October 12^th^ at 0.04^°^ and daily resolution. From Fig. 10 it can be seen that compared to PERSIANN-CCS-CDR-CPC and STAGE IV, PERSIANN-CCS-CDR-B1 images appear coarser. This is because PERSIANN-CCS-CDR-B1 has fewer samples in each time step, and the PERSIANN-CCS-CDR algorithm was tailored to CPC-4KM data and then applied to GridSat-B1 data. Furthermore, both PERSIANN-CCS-CDR-CPC and PERSIANN-CCS-CDR-B1 do a very good job of capturing the event.

**Fig. 11:**
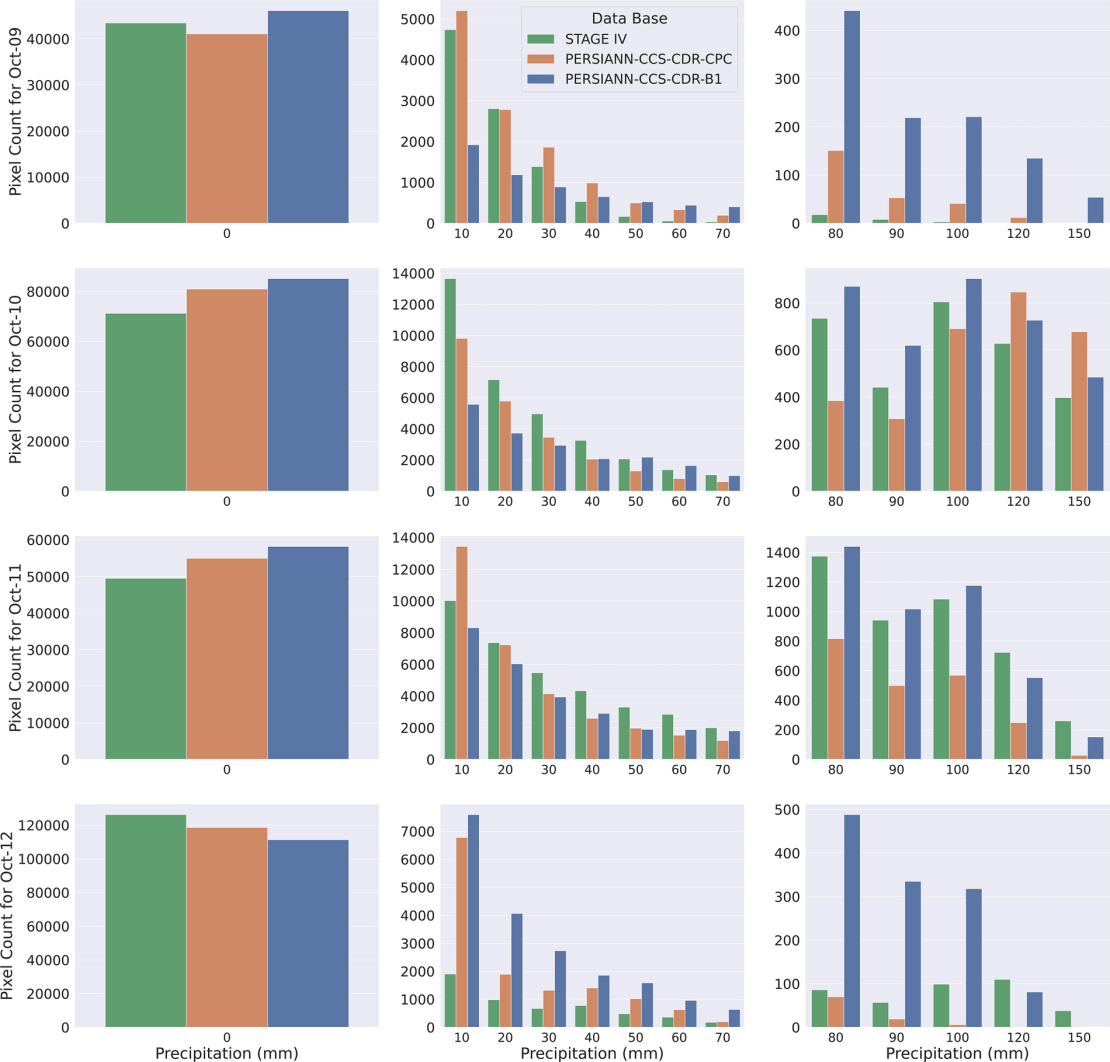
Bin plots for Hurricane Michael precipitation at 0.04^°^ and daily resolution for STAGE IV in green, PERSIANN-CCS-CDR-CPC in orange, and PERSIANN-CCS-CDR-B1 in blue.

While visual inspection of snapshots provides valuable insights, assessing the distribution of precipitation requires us to look at Fig. 11, which presents bin plots for Hurricane Michael at 0.04^°^ and daily resolution. We can see that estimations of PERSIANN-CCS-CDR-CPC and PERSIANN-CCS-CDR-B1 have a high aggrement with estimations of STAGE IV, however they both have higher no-rain pixels compared to STAGE IV, which means that they underestimate the extent of the hurricane (the leftmost column). It is also seen that PERSIANN-CCS-CDR-CPC has a better agreement with STAGE IV than PERSIANN-CCS-CDR-B1. Furthermore, from the rightmost column, it seems that PERSIANN-CCS-CDR-B1 occasionally assigns unrealistically high values to some pixels, primarily due to a downsampling issue in the dataset. Additionally, because PERSIANN-CCS-CDR-B1 uses only one input every three hours (compared to one input every 30 minutes for PERSIANN-CCS-CDR-CPC), its ability to capture fast-evolving events like hurricanes is inherently limited. Consequently, it fails to represent the full spatial extent of such events; as a result, it compensates by distributing the water budget over fewer pixels.

**Fig. 12:**
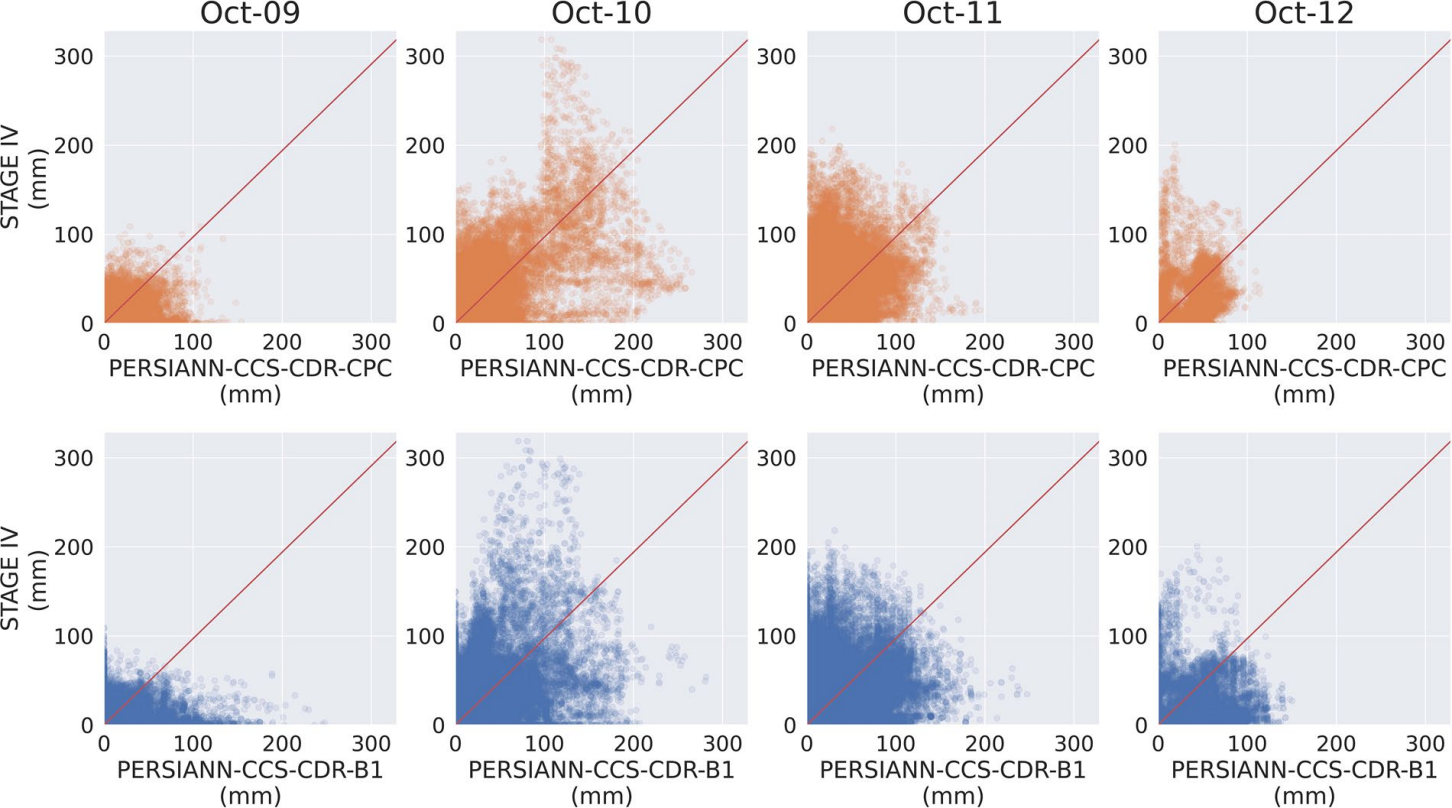
Scatter plot of STAGE IV compared to PERSIANN-CCS-CDR-CPC (top), and PERSIANN-CCS-CDR-B1 (bottom) for Hurricane Michael at 0.04^°^ and daily resolution.

To further compare the distribution of precipitation between the datasets, we turn to Fig. 12, which shows the scatter plot of STAGE IV, PERSIANN-CCS-CDR-CPC, and PERSIANN-CCS-CDR-B1 for Hurricane Michael at 0.04^°^ and daily resolution. The sharp vertical surge of points on October 10^th^ indicates that both PERSIANN-CCS-CDR-CPC and PERSIANN-CCS-CDR-B1 failed to capture the intense precipitation on that day. This aligns with the findings of^33^, which suggest that satellite precipitation products struggle to detect extremely high precipitation rates. Furthermore, when we look at the PERSIANN-CCS-CDR-B1 plots, we see that PERSIANN-CCS-CDR-B1 records some high-value pixels; however, these do not align with the corresponding STAGE IV values. This indicates that while PERSIANN-CCS-CDR-B1 records extreme values, they are not a result of its accuracy but rather an issue with the downsampling process. If overlooked, this could lead to incorrect interpretations.

**Fig. 13:**
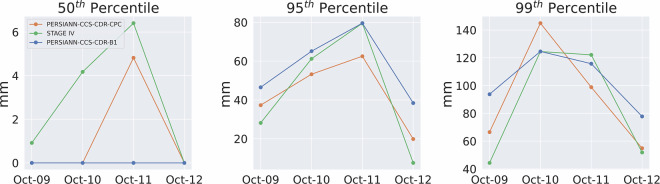
Plots for 50^th^ (left), 95^th^ (middle), and 99^th^ (right) percentiles for STAGE IV in green, PERSIANN-CCS-CDR-CPC in orange, and PERSIANN-CCS-CDR-B1 for blue for Hurricane Michael at 0.04^°^ and daily resolution.

Furthermore, Fig. 13 shows the value of the 50^th^, 95^th^ and 99^th^ percentiles for STAGE IV, PERSIANN-CCS-CDR-CPC, and PERSIANN-CCS-CDR-B1 for Hurricane Michael at 0.04^°^ and daily resolution. As can be seen in the left panel, compared to STAGE IV, PERSIANN-CCS-CDR-CPC and, to a greater extent, PERSIANN-CCS-CDR-B1 are unable to estimate the light rain, which is a well-known issue in satellite-based estimation methods^34^. As a result, they are unable to capture the full extent of the event. Also, from the middle and right panels, It can be seen that both datasets perform very similarly to STAGE IV. However, one might conclude that the PERSIANN-CCS-CDR-B1 dataset appears to better capture extreme and very extreme values. As noted earlier, though, these higher values result from downsampling issues in PERSIANN-CCS-CDR-B1 and do not represent the true skill of the dataset.

**Fig. 14:**
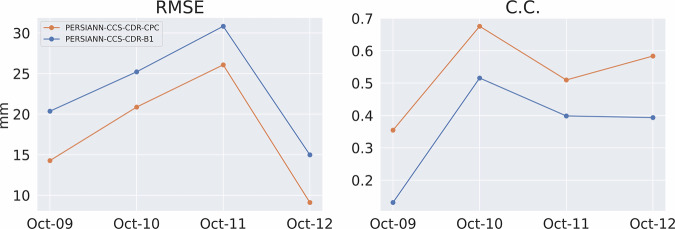
Plot of RMSE (left) and C.C. (right) for PERSIANN-CCS-CDR-CPC in orange and PERSIANN-CCS-CDR-B1 in blue compared to STAGE IV for Hurricane Michael at 0.04^°^ and daily resolution.

**Fig. 15:**
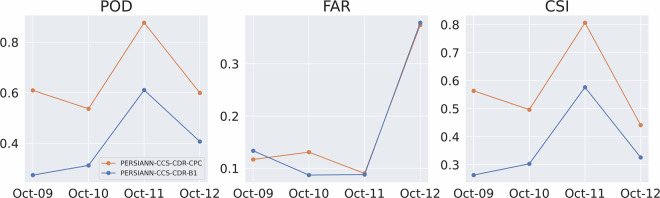
Plot of POD (left), FAR (middle), and CSI (right) for PERSIANN-CCS-CDR-CPC in orange and PERSIANN-CCS-CDR-B1 in blue compared to STAGE IV for Hurricane Michael at 0.04^°^ and daily resolution.

Figure 14 shows the RMSE and CC for the event at 0.04^°^ and daily resolution, and Fig. 15 shows the POD, FAR, and CSI for the event at 0.04^°^ and daily resolution. These figures once again tell the same story: both PERSIANN-CCS-CDR-CPC and PERSIANN-CCS-CDR-B1 overally perform similarly to STAGE IV, but PERSIANN-CCS-CDR-CPC consistently outperforms PERSIANN-CCS-CDR-B1. From Fig. 14, we can conclude that PERSIANN-CCS-CDR-CPC has a lower RMSE and higher C.C. than PERSIANN-CCS-CDR-B1 when compared to STAGE IV. Also in Fig. 15 it can be seen that PERSIANN-CCS-CDR-CPC outperforms the PERSIANN-CCS-CDR-B1 in all metrics.

#### Hurricane Michael 3-hourly Analysis at 0.04^°^ Resolution

**Fig. 16:**
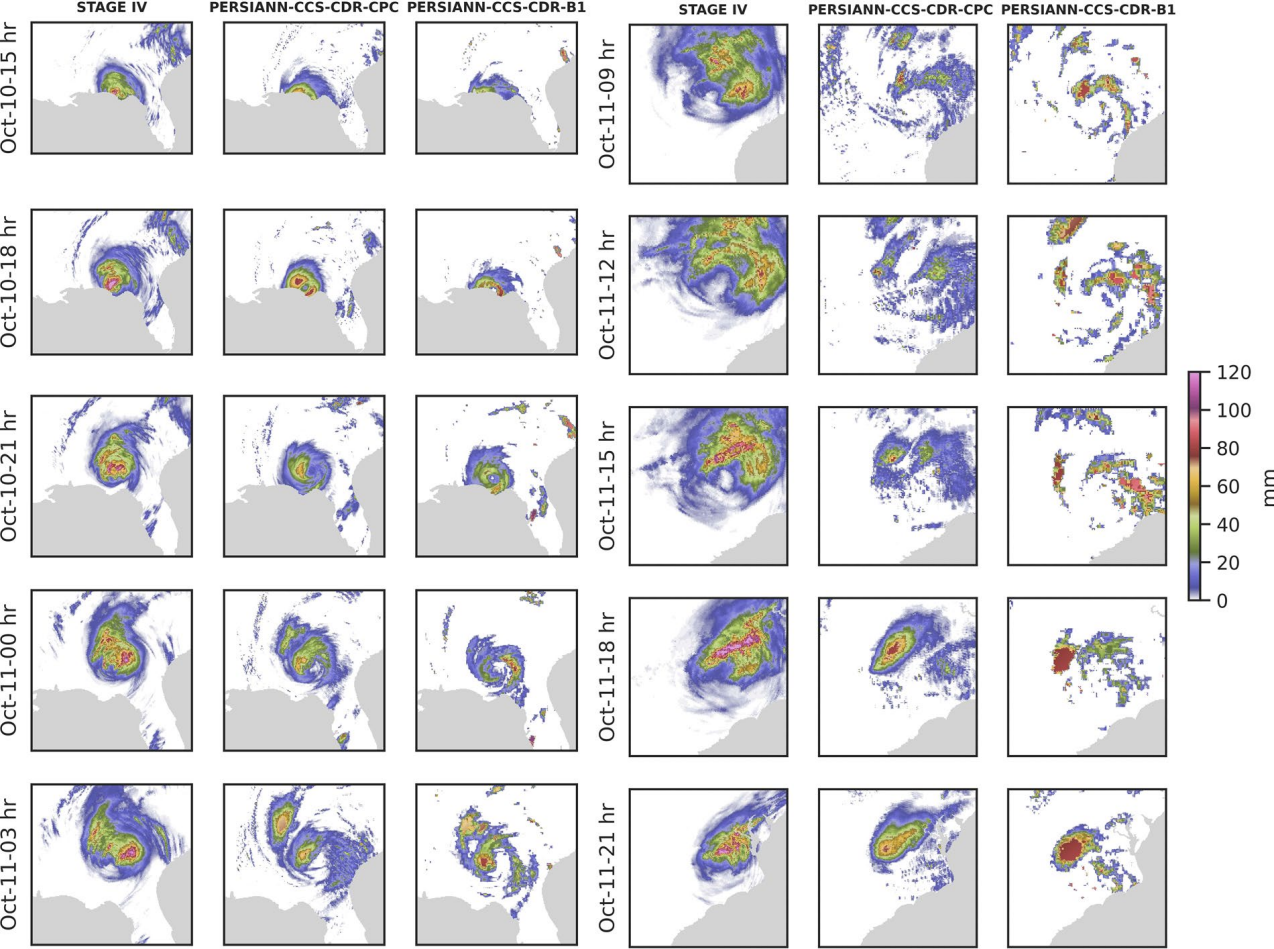
Snapshots of Hurricane Michael according to STAGE IV, PERSIANN-CCS-CDR-CPC, and, PERSIANN-CCS-CDR-B1 datasets at 0.04^°^ and 3-hour resolution.

After establishing a clear picture of the datasets’ performance at high spatial resolution, we now shift our focus to temporal resolution by conducting the same analyses at a 3-hourly resolution. Figure 16 shows 3-hourly accumulated snapshots of hurricane Michael at 0.04^°^ resolution; to save space, not all snapshots are shown here. Additionally, since the study focuses only on land, in cases in which snapshots include portions of the open ocean, the frames were cropped to create a tight layout, as a result, not all frames have the same dimensions, and some might appear as if they are zoomed in or out, but this is only an effect of the cropping. This effect is further amplified by the fact that the storm radius, and hence the frame bounds, changes at each time step. As observed, both datasets have a high aggrement with STAGE IV, however PERSIANN-CCS-CDR-CPC performs better in capturing the events compared to PERSIANN-CCS-CDR-B1. This is primarily because the PERSIANN-CCS-CDR algorithm is tailored to PERSIANN-CCS-CDR-CPC input data, and PERSIANN-CCS-CDR-CPC benefits from using six inputs every three hours, whereas PERSIANN-CCS-CDR-B1 relies on only one input during the same period. Overall, both PERSIANN-CCS-CDR-B1 and PERSIANN-CCS-CDR-CPC demonstrated strong performance in capturing the extent of the hurricane up to October 11^th^ at 6 hr. After that, the hurricane structure in PERSIANN-CCS-CDR-CPC and PERSIANN-CCS-CDR-B1 starts to collapse. This problem is due to the limitation in the PERSIANN algorithm; as hurricanes move inland and weaken, although they can still produce significant amounts of rainfall, they often experience a temperature rise. However, since the PERSIANN algorithm only uses the IR temperature of the cloud top to estimate precipitation, it’s unable to estimate the precipitation under these circumstances.

**Fig. 17:**
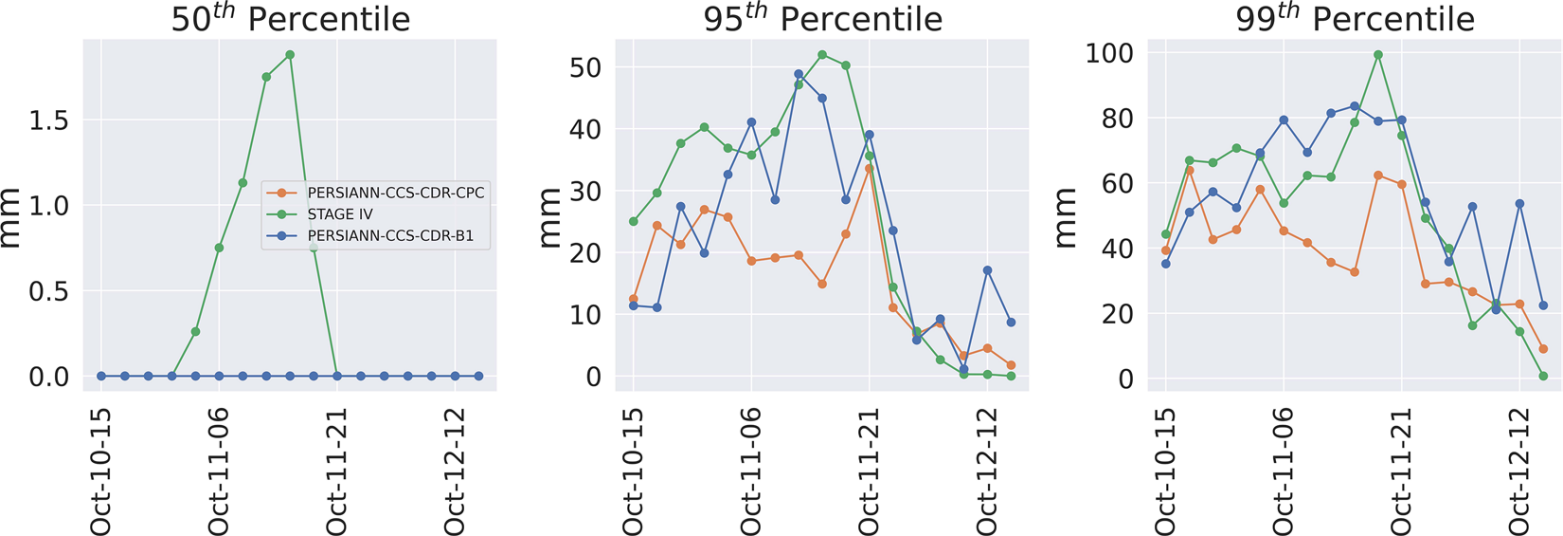
Plots for 50^th^ (left), 95^th^ (middle) and 99^th^ (right), percentile for STAGE IV in green, PERSIANN-CCS-CDR-CPC in orange, and PERSIANN-CCS-CDR-B1 in blue for Hurricane Michael at 0.04^°^ and 3-hour resolution. Note that in the 50^th^ percentile plot, the values for PERSIANN-CCS-CDR-CPC completely overlap with those of PERSIANN-CCS-CDR-B1.

Figure 17 shows the 50^th^, 95^th^, and 99^th^ percentile values for the entire event at 0.04^°^ and 3-hourly resolution starting at October 10^th^ 3 PM until October 12^th^ 1 PM. The same trend as Fig. 13 can be seen here: Firstly, PERSIANN-CCS-CDR-CPC and PERSIANN-CCS-CDR-B1 are unable to capture the light rain (left figure). Secondly, although both datasets closely match with STAGE IV, PERSIANN-CCS-CDR-CPC and PERSIANN-CCS-CDR-B1 fail to fully capture the extreme values, and as mentioned earlier the higher values for the PERSIANN-CCS-CDR-B1 95^th^ and 99^th^ percentile compared to that of PERSIANN-CCS-CDR-CPC, are the result of a downsampling problem and should not be mistaken for higher skill of the PERSIANN-CCS-CDR-B1 product compared to PERSIANN-CCS-CDR-CPC.

**Fig. 18:**
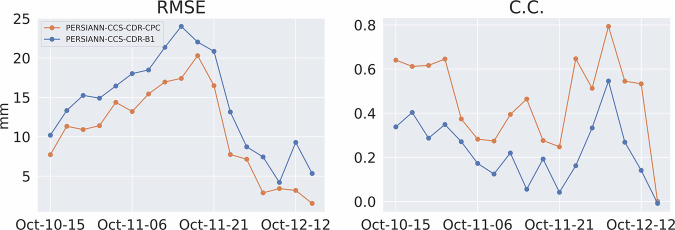
Plot for RMSE (left) and C.C. (right) for PERSIANN-CCS-CDR-CPC in orange and PERSIANN-CCS-CDR-B1 in blue compared to STAGE IV for Hurricane Michael at 0.04^°^ and 3-hourly resolution.

Figure 18 shows the RMSE and C.C. for the entire event at 0.04^°^ and 3-hourly resolution. As already seen at daily resolution, although both datasets perform very well compared to STAGE IV, the PERSIANN-CCS-CDR-CPC outperforms the PERSIANN-CCS-CDR-B1 in both of these tests. It’s important to note that the values for the final snapshot (October 12^th^, 13hr) correspond to a time when the storm affected only a very small portion of CONUS. As a result, these values should not be considered representative. However, they are included in the results for consistency.

**Fig. 19:**
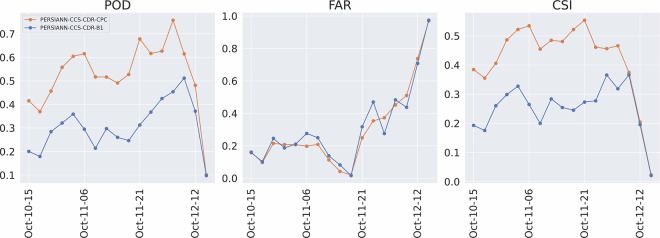
Plots for POD (left), FAR (middle), and CSI (right) for PERSIANN-CCS-CDR-CPC in orange and PERSIANN-CCS-CDR-B1 in blue compared to STAGE IV for Hurricane Michael at 0.04^°^ and 3-hourly resolution.

Figure 19 shows the POD, FAR, and CSI for the entire event at 3-hourly resolutions. Both datasets have the same performance in FAR. However, PERSIANN-CCS-CDR-CPC better captures the extent of the event, which leads to a better POD and, naturally, a better CSI.

#### Hurricane Michael Daily Analysis at 0.25^°^ Resolution

**Fig. 20:**
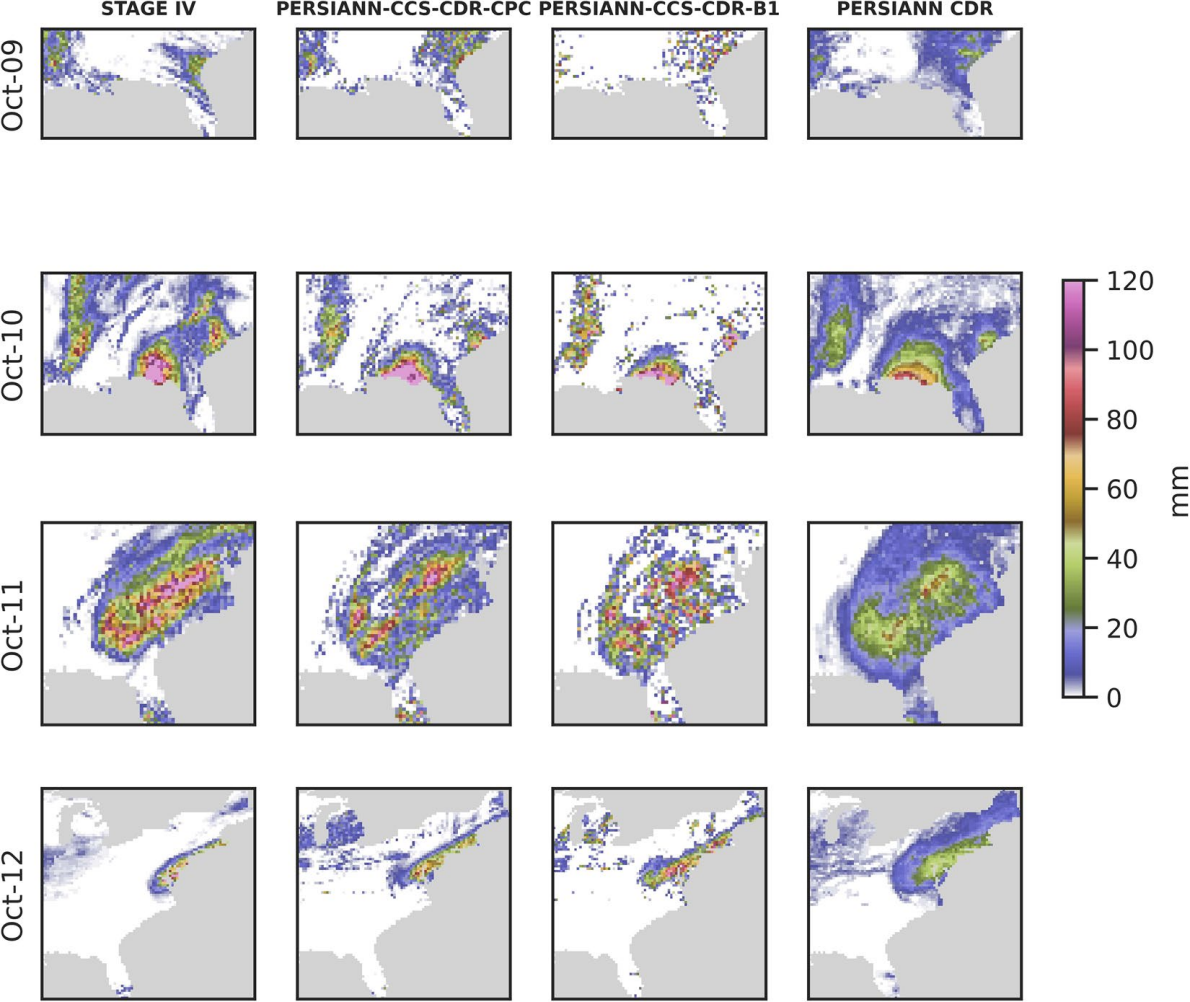
Snapshots of hurricane Michael according to STAGE IV, PERSIANN-CCS-CDR-CPC, PERSIANN-CCS-CDR-B1, and PERSIANN-CDR at 0.25^°^ and daily resolution.

**Fig. 21:**
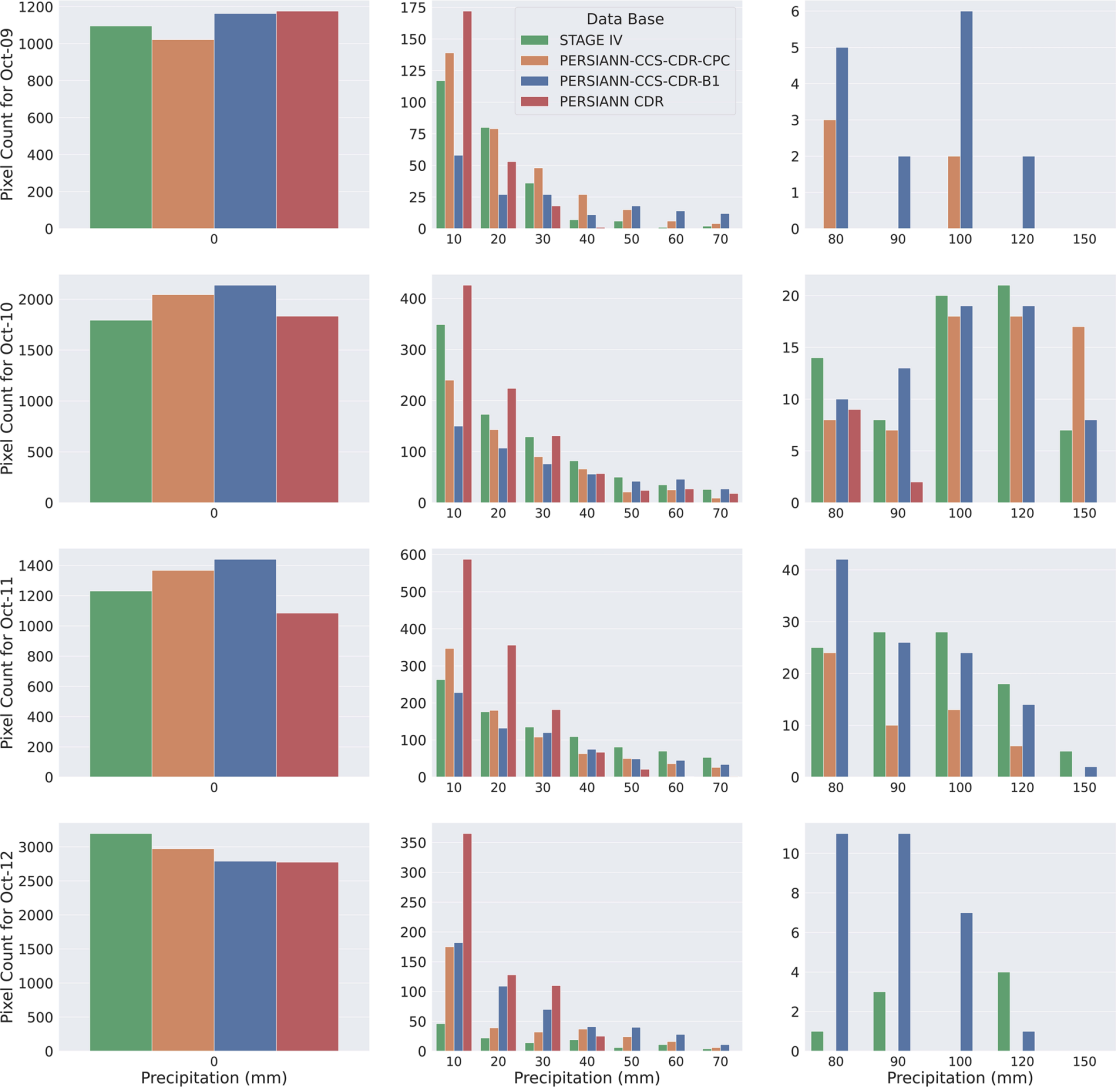
Bin plots for hurricane Michael at 0.25^°^ and daily resolution for STAGE IV in green, PERSIANN-CCS-CDR-CPC in orange, PERSIANN-CCS-CDR-B1 in blue, and PERSIANN-CDR in red.

In the previous sections, we demonstrated that PERSIANN-CCS-CDR-CPC outperforms PERSIANN-CCS-CDR-B1 when compared to STAGE IV. However, it is important to note that, despite its relatively lower performance compared to PERSIANN-CCS-CDR-CPC, PERSIANN-CCS-CDR-B1 still exhibits skill, and it’s a valuable product. To further illustrate this, the same analysis was conducted at a 0.25^°^ resolution, with the inclusion of PERSIANN-CDR. Figure 20 shows a snapshot, and Fig. 21 shows the bin plot of precipitation for Hurricane Michael at 0.25^°^ and daily resolution with the inclusion of the PERSIANN-CDR dataset. From Fig. 20 and the left column of Fig. 21, we can see that PERSIANN-CDR has the least number of no-rain pixels and higher pixel counts for low precipitation rates (10 mm, 20 mm, 30 mm). This means that PERSIANN-CDR overestimates light rain and the extent of the event. More importantly, PERSIANN-CDR barely detects rain above the 70 mm threshold, which means it’s unable to detect extreme values.

**Fig. 22:**
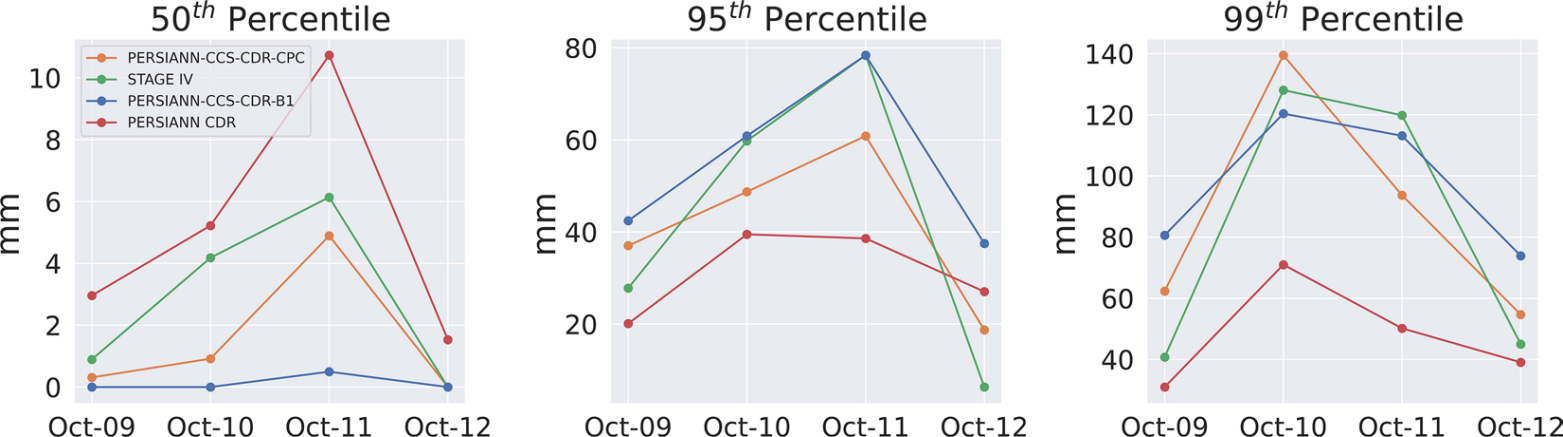
Plots for 50^th^ (left), 95^th^ (middle) and 99^th^ percentile (right) for STAGE IV in green, PERSIANN-CCS-CDR-CPC in orange, PERSIANN-CCS-CDR-B1 in blue, and PERSIANN-CDR in red for hurricane Michael at 0.25^°^ and daily resolution.

Figure 22 shows the 50^th^, 95^th^, and 99^th^ percentile plots for STAGE IV, PERSIANN-CCS-CDR-CPC, PERSIANN-CCS-CDR-B1, and PERSIANN-CDR for Hurricane Michael at 0.25^°^ and daily resolution. Here we can also see that PERSIANN-CDR overestimates the light rain (left panel), and it has the worst performance compared to STAGE IV at estimating the 95^th^ and 99^th^ percentile values. Concerning PERSIANN-CCS-CDR-CPC and PERSIANN-CCS-CDR-B1, the same pattern seen at 0.04^°^ is also observed at 0.25^°^ resolution.

**Fig. 23:**
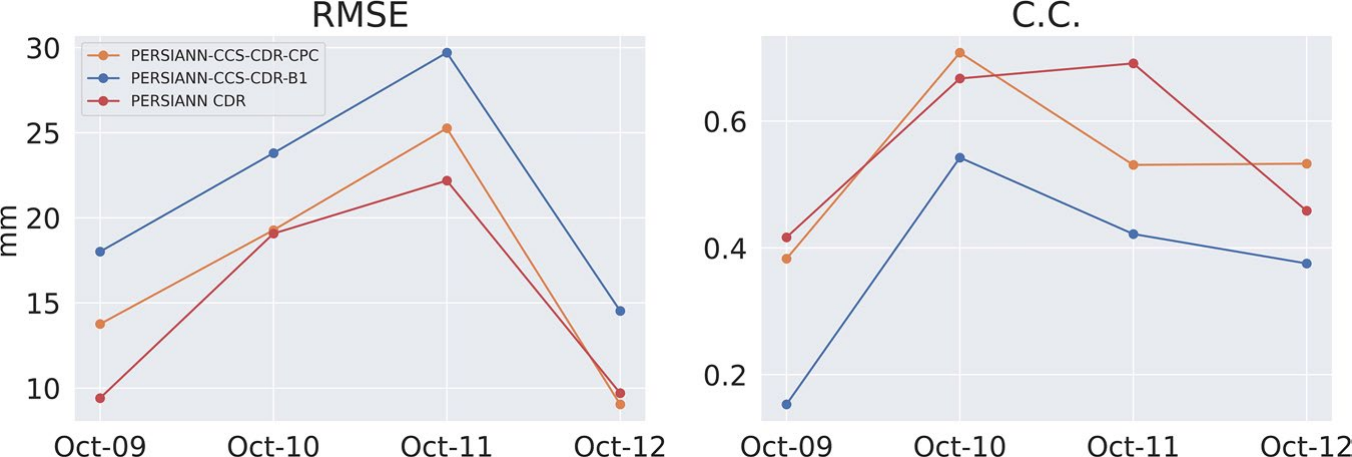
Plot for RMSE (left) and C.C. (right) for PERSIANN-CCS-CDR-CPC in orange, PERSIANN-CCS-CDR-B1 in blue, and PERSIANN-CDR in red compared to STAGE IV for Hurricane Michael at 0.25^°^ and daily resolution.

**Fig. 24:**
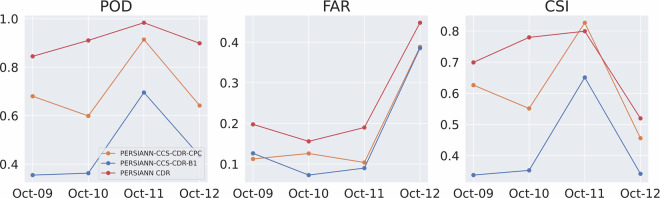
Plots for POD (left), FAR (middle), and CSI (right) for PERSIANN-CCS-CDR-CPC in orange, PERSIANN-CCS-CDR-B1 in blue, and PERSIANN-CDR in red compared to STAGE IV for Hurricane Michael at 0.25^°^ and daily resolution.

Figure 23 shows the RMSE and C.C., and Fig. 24 shows the POD, FAR, and CSI for hurricane Michael at 0.25^°^ and daily resolution. From these figures, we can see that despite the shortcomings mentioned above, PERSIANN-CDR has better RMSE, C.C., and CSI values than PERSIANN-CCS-CDR-B1 and PERSIANN-CCS-CDR-CPC, which further emphasizes the superiority of PERSIANN-CDR at low spatial and temporal resolutions. On the other hand, although PERSIANN-CDR has the highest POD, it also has the highest FAR, which is a result of PERSIANN-CDR’s overestimation of light rain and event extent.

#### Upper Midwest Flood of 2024

**Fig. 25:**
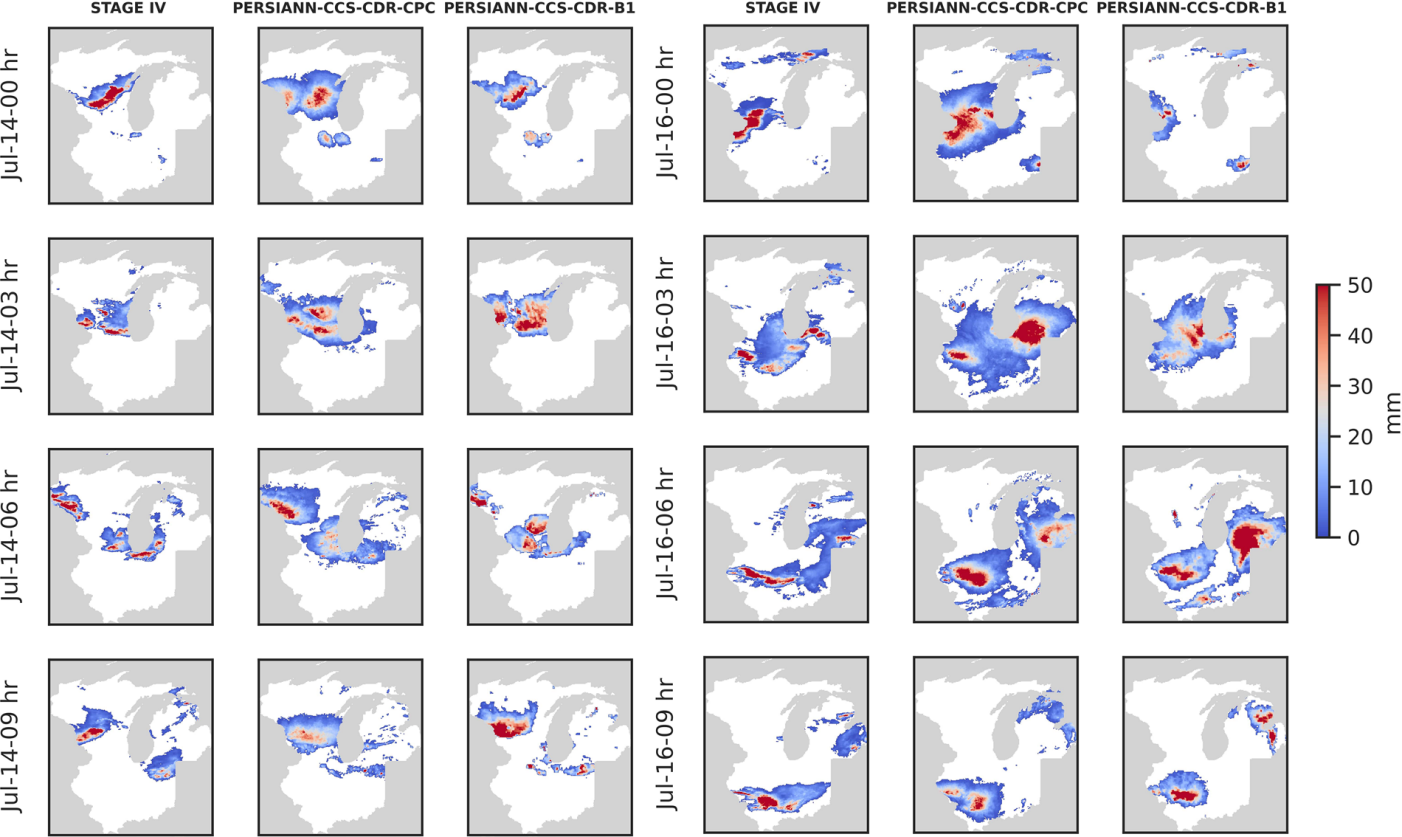
Snapshots of Upper Midwest Flood according to STAGE IV (left), PERSIANN-CCS-CDR-CPC (middle), and, PERSIANN-CCS-CDR-B1 (right) datasets at 0.04^°^ and 3-hour resolution.

To further assess the performance of the datasets at high spatiotemporal resolution, we examined the severe weather event over the Upper Midwest from July 13^th^, 15:00, to July 16^th^, 18:00, 2024. Figure 25 shows few snapshots of this event as captured by STAGE IV, PERSIANN-CCS-CDR-CPC, and PERSIANN-CCS-CDR-B1. From these snapshots, it is evident that both PERSIANN-CCS-CDR-CPC and PERSIANN-CCS-CDR-B1 show strong agreement with STAGE IV observations.

**Fig. 26:**
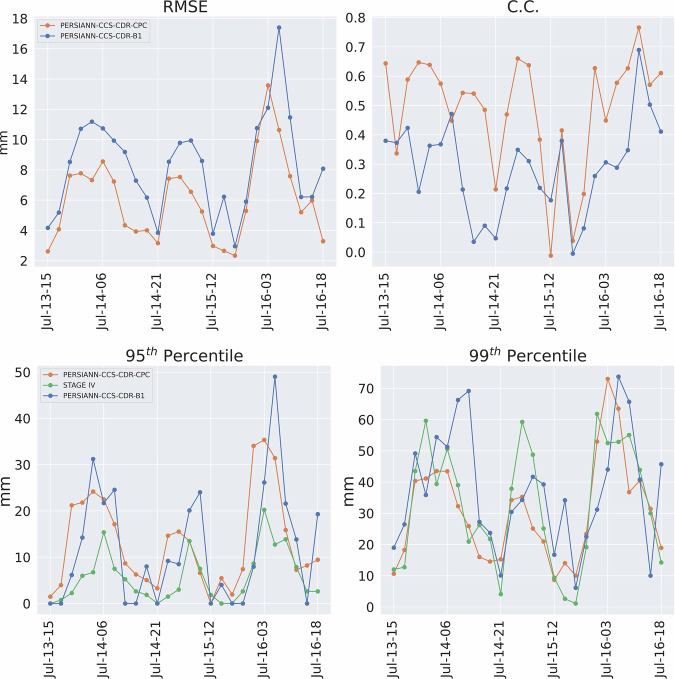
Plots for RMSE (top left), C.C. (top right) for PERSIANN-CCS-CDR-CPC in orange and PERSIANN-CCS-CDR-B1 in blue compared to STAGE IV, and 95^th^ percentile (bottom left), and 99^th^ percentile for STAGE IV in green, PERSIANN-CCS-CDR-CPC in orange, and PERSIANN-CCS-CDR-B1 in blue for Upper Midwest Flood at 0.04^°^ and 3-hour resolution.

Furthermore, Fig. 26 shows the RMSE, C.C. for PERSIANN-CCS-CDR-CPC and PERSIANN-CCS-CDR-B1 compared to STAGE IV, along with the 95^th^ and 99^th^ percentile precipitation values for STAGE IV, PERSIANN-CCS-CDR-CPC, and PERSIANN-CCS-CDR-B1. The results show that both datasets have a close performance to STAGE IV, with PERSIANN-CCS-CDR-CPC consistently outperforming PERSIANN-CCS-CDR-B1. Additionally, periods of drastic degradation in C.C. are seen in both datasets, which coincide with cooling intervals between the two storm peaks (evident from near-zero 95^th^ percentile values). Because in instances of almost no precipitation even slight misalignments between observations can drastically affect the C.C. these results are expected under such conditions. In short, both PERSIANN-CCS-CDR-CPC and PERSIANN-CCS-CDR-B1 are consistent datasets and are suitable for use in both event-based analyses and climate studies. It should also be noted that the two subproducts differ in performance during their overlapping period. However, the authors would also like to emphasize that:

- PERSIANN-CCS-CDR Version 2.0 is designed for high spatial-temporal analysis and delivers optimal performance when applied to tasks requiring such resolutions. For other purposes, authors suggest users to use other products such as PERSIANN-CDR, and PDIR-Now.
- When using PERSIANN-CCS-CDR Version 2.0 users are confronted with a key decision: either to opt for the PERSIANN-CCS-CDR-B1 dataset, which offers a longer data record but lower performance, or to choose the PERSIANN-CCS-CDR-CPC dataset and sacrifice record period for the sake of improved performance.
- When choosing between PERSIANN-CCS-CDR-CPC and PERSIANN-CCS-CDR-B1, it is best to prioritize the PERSIANN-CCS-CDR-CPC dataset. The PERSIANN-CCS-CDR-B1 dataset should be used primarily when the analysis focuses on extreme precipitation events prior to March 2000 or when over 40 years of high temporal and spatial resolution data are required.

## Data Availability

PERSIANN-CCS-CDR V2.0 is a high spatial-temporal resolution precipitation estimation dataset that consists of two sub-products: PERSIANN-CCS-CDR-CPC, available from March 2000, and PERSIANN-CCS-CDR-B1, available from 1983. Both datasets have a 0.04^°^ spatial and 3-hourly temporal resolution, with coverage from 60^°^S to 60^°^N. PERSIANN-CCS-CDR V2.0 is available publicly through the CHRS Data Portal (https://chrsdata.eng.uci.edu/) at 3-hourly, 6-hourly, daily, monthly, and yearly time steps. The data is also available through the CHRS FTP website (https://persiann.eng.uci.edu/CHRSdata/PCCSCDR_B1/and https://persiann.eng.uci.edu/CHRSdata/PCCSCDR_CPC/) in binary format.

## Code availability

The Python scripts used to generate the figures and perform the analysis are available in a public GitHub repository (https://github.com/bolbolim/PERSIANN-CCS-CDR-V02). Users are responsible for downloading the necessary data to run the code. For any technical issues related to data access or code execution, please contact the corresponding author.

## References

[1] Habib, E., Haile, A. T., Tian, Y. & Joyce, R. J. Evaluation of the high-resolution cmorph satellite rainfall product using dense rain gauge observations and radar-based estimates. *Journal of Hydrometeorology* **13**, 1784–1798 (2012).

[2] Nguyen, P. *et al*. Persiann dynamic infrared–rain rate (pdir-now): A near-real-time, quasi-global satellite precipitation dataset. *Journal of hydrometeorology* **21**, 2893–2906 (2020). 34158807 10.1175/jhm-d-20-0177.1 PMC8216223

[3] Behrangi, A. *et al*. Persiann-msa: A precipitation estimation method from satellite-based multispectral analysis. *Journal of Hydrometeorology* **10**, 1414–1429 (2009).

[4] Marzano, F. S., Palmacci, M., Cimini, D., Giuliani, G. & Turk, F. J. Multivariate statistical integration of satellite infrared and microwave radiometric measurements for rainfall retrieval at the geostationary scale. *IEEE transactions on Geoscience and remote sensing* **42**, 1018–1032 (2004).

[5] Kidd, C. & Levizzani, V. Status of satellite precipitation retrievals. *Hydrology and Earth System Sciences* **15**, 1109–1116 (2011).

[6] Rouzegari, N. *et al*. Passive microwave imagers, their applications, and benefits: A review. *Remote Sensing* **17** (2025).

[7] Grecu, M., Olson, W. S. & Anagnostou, E. N. Retrieval of precipitation profiles from multiresolution, multifrequency active and passive microwave observations. *Journal of Applied Meteorology and Climatology* **43**, 562–575 (2004).

[8] Funk, C. *et al*. The climate hazards infrared precipitation with stations—a new environmental record for monitoring extremes. *Scientific data* **2**, 1–21 (2015). 10.1038/sdata.2015.66 PMC4672685 26646728

[9] Janowiak, J. E., Joyce, R. J. & Yarosh, Y. A real-time global half-hourly pixel-resolution infrared dataset and its applications. *Bulletin of the American Meteorological Society* **82**, 205–218 (2001).

[10] Knapp, K. R. *et al*. Globally gridded satellite observations for climate studies. *Bulletin of the American Meteorological Society* **92**, 893–907 (2011).

[11] NOAA. Gridsat-b1 f.a.q. NOAA National Centers for Environmental Information Accessed: 2024-8-14 (2024).

[12] Nguyen, P. *et al*. The chrs data portal, an easily accessible public repository for persiann global satellite precipitation data. *Scientific data* **6**, 1–10 (2019). 30620343 10.1038/sdata.2018.296 PMC6326112

[13] Hsu, K.-l, Gao, X., Sorooshian, S. & Gupta, H. V. Precipitation estimation from remotely sensed information using artificial neural networks. *Journal of Applied Meteorology and Climatology* **36**, 1176–1190 (1997).

[14] Hong, Y., Hsu, K.-L., Sorooshian, S. & Gao, X. Precipitation estimation from remotely sensed imagery using an artificial neural network cloud classification system. *Journal of Applied Meteorology* **43**, 1834–1853 (2004).

[15] Ashouri, H. *et al*. Persiann-cdr: Daily precipitation climate data record from multisatellite observations for hydrological and climate studies. *Bulletin of the American Meteorological Society* **96**, 69–83 (2015).

[16] Huffman, G. J. *et al*. Integrated multi-satellite retrievals for the global precipitation measurement (gpm) mission (imerg). *Satellite precipitation measurement: Volume 1* 343–353 (2020).

[17] Sadeghi, M. *et al*. Persiann-ccs-cdr, a 3-hourly 0.04 global precipitation climate data record for heavy precipitation studies. *Scientific Data* **8**, 157 (2021). 34162874 10.1038/s41597-021-00940-9 PMC8222311

[18] CHRS. Center for hydrometeorology and remote sensing (chrs) data portal. https://chrsdata.eng.uci.edu/ (2024).

[19] CHRS. Precipitation estimation from remotely sensed information using artificial neural networks-cloud classification system-climate data record version 2.0. 10.5061/dryad.n8pk0p38q (2024).

[20] Du, J. Ncep/emc 4km gridded data (grib) stage iv data. version 1.0. UCAR/NCAR - Earth Observing Laboratory. 10.5065/D6PG1QDD (2011).

[21] Nelson, B. R., Prat, O. P., Seo, D.-J. & Habib, E. Assessment and implications of ncep stage iv quantitative precipitation estimates for product intercomparisons. *Weather and Forecasting* **31**(2), 371–394 (2016).

[22] Kursinski, A. L. & Mullen, S. L. Spatiotemporal variability of hourly precipitation over the eastern contiguous united states from stage iv multisensor analyses. *Journal of Hydrometeorology* **9**, 3–21 (2008).

[23] Hou, D. *et al*. Climatology-calibrated precipitation analysis at fine scales: Statistical adjustment of stage iv toward cpc gauge-based analysis. *Journal of Hydrometeorology* **15**(6), 2542–2557 (2014).

[24] Maddox, R. A., Zhang, J., Gourley, J. J. & Howard, K. W. Weather radar coverage over the contiguous united states. *Weather and forecasting* **17**(4), 927–934 (2002).

[25] NOAA NESDIS Severe thunderstorms race through the midwest. NESDIS, NOAA Accessed: 2025-10-21 (2024).

[26] NOAA NCEI U.s. 2024 billion-dollor weather and climate disasters. NCEI, NOAA Accessed: 2025-10-21 (2024).

[27] Knapp, K. R., Kruk, M. C., Levinson, D. H., Diamond, H. J. & Neumann, C. J. The international best track archive for climate stewardship (ibtracs) unifying tropical cyclone data. *Bulletin of the American Meteorological Society* **91**(3), 363–376 (2010).

[28] Gahtan, J. *et al*. International best track archive for climate stewardship (ibtracs) project, version 4r01. [indicate subset used]. NOAA National Centers for Environmental Information. 10.25921/82ty-9e16 (2024).

[29] U.S. Census Bureau Cartographic boundary files - shapefile. U.S. Census Bureau Accessed: 2024-8-14 (2024).

[30] Lehner, B. & Grill, G. Global river hydrography and network routing: baseline data and new approaches to study the world’s large river systems. *Hydrological Processes* **27**, 2171–2186 (2013).

[31] de Medeiros, F. J., de Oliveira, C. P. & Avila-Diaz, A. Evaluation of extreme precipitation climate indices and their projected changes for brazil: From cmip3 to cmip6. *Weather and Climate Extremes* **38**, 100511 (2022).

[32] Soltani, M. *et al*. Assessment of climate variations in temperature and precipitation extreme events over iran. *Theoretical and Applied Climatology* **126**, 775–795 (2016).

[33] Mehran, A. & AghaKouchak, A. Capabilities of satellite precipitation datasets to estimate heavy precipitation rates at different temporal accumulations. *Hydrological Processes* **28**, 2262–2270 (2014).

[34] Sorooshian, S. *et al*. Advanced concepts on remote sensing of precipitation at multiple scales. *Bulletin of the American Meteorological Society* **92**, 1353–1357 (2011).

